# Potent Killing of Pseudomonas aeruginosa by an Antibody-Antibiotic Conjugate

**DOI:** 10.1128/mBio.00202-21

**Published:** 2021-06-01

**Authors:** Kimberly K. Kajihara, Homer Pantua, Hilda Hernandez-Barry, Meredith Hazen, Kiran Deshmukh, Nancy Chiang, Rachana Ohri, Erick R. Castellanos, Lynn Martin, Marissa L. Matsumoto, Jian Payandeh, Kelly M. Storek, Kellen Schneider, Peter A. Smith, Michael F. T. Koehler, Siao Ping Tsai, Richard Vandlen, Kelly M. Loyet, Gerald Nakamura, Thomas Pillow, Dhaya Seshasayee, Sharookh B. Kapadia, Wouter L. W. Hazenbos

**Affiliations:** a Department of Infectious Diseases, Genentech, Inc., South San Francisco, California, USA; b Department of Biochemical and Cellular Pharmacology, Genentech, Inc., South San Francisco, California, USA; c Department of Antibody Engineering, Genentech, Inc., South San Francisco, California, USA; d Department of Protein Chemistry, Genentech, Inc., South San Francisco, California, USA; e Department of Structural Biology, Genentech, Inc., South San Francisco, California, USA; f Department of BioMolecular Resources, Genentech, Inc., South San Francisco, California, USA; g Department of Medicinal Chemistry; Genentech, Inc., South San Francisco, California, USA

**Keywords:** antibody-antibiotic conjugate, *Pseudomonas aeruginosa*, antibiotics, macrophage

## Abstract

Pseudomonas aeruginosa causes life-threatening infections that are associated with antibiotic failure. Previously, we identified the antibiotic G2637, an analog of arylomycin, targeting bacterial type I signal peptidase, which has moderate potency against P. aeruginosa. We hypothesized that an antibody-antibiotic conjugate (AAC) could increase its activity by colocalizing P. aeruginosa bacteria with high local concentrations of G2637 antibiotic in the intracellular environment of phagocytes. Using a novel technology of screening for hybridomas recognizing intact bacteria, we identified monoclonal antibody 26F8, which binds to lipopolysaccharide O antigen on the surface of P. aeruginosa bacteria. This antibody was engineered to contain 6 cysteines and was conjugated to the G2637 antibiotic via a lysosomal cathepsin-cleavable linker, yielding a drug-to-antibody ratio of approximately 6. The resulting AAC delivered a high intracellular concentration of free G2637 upon phagocytosis of AAC-bound P. aeruginosa by macrophages, and potently cleared viable P. aeruginosa bacteria intracellularly. The molar concentration of AAC-associated G2637 antibiotic that resulted in elimination of bacteria inside macrophages was approximately 2 orders of magnitude lower than the concentration of free G2637 required to eliminate extracellular bacteria. This study demonstrates that an anti-P. aeruginosa AAC can locally concentrate antibiotic and kill P. aeruginosa inside phagocytes, providing additional therapeutic options for antibiotics that are moderately active or have an unfavorable pharmacokinetics or toxicity profile.

## INTRODUCTION

Infections caused by Gram-negative Pseudomonas aeruginosa bacteria, in particular pulmonary and bloodstream infections, are associated with antibiotic failure and high mortality (1–4), which is further complicated by the emerging spread of multidrug-resistant (MDR) P. aeruginosa (3–6). In 2017, the World Health Organization categorized MDR P. aeruginosa as a “critical threat” pathogen (7). A 2019 CDC report estimated 32,600 cases of infection and 2,700 deaths in the United States per year caused by MDR P. aeruginosa infections (8). During chronic infections, P. aeruginosa undergoes significant morphologic and phenotypic changes, contributing to reduced efficacy of current antibiotics. P. aeruginosa isolates from cystic fibrosis patients are highly adapted to their local environment and even diversify within different regions of the lung with respect to antibiotic sensitivity (9). Antibiotics that are clinically used for the treatment of P. aeruginosa infections are often dose limited (3) and may not achieve sufficient local concentrations at the site of infection. Identification of strategies to increase local antibiotic concentrations may enable more effective clearance.

Antibody-drug conjugates (ADC) represent a clinically proven approach to selectively deliver high concentrations of cytotoxic drugs to the local environment of tumor cells (10, 11). Based on this technology, we previously generated an antibody-antibiotic conjugate (AAC) molecule against the Gram-positive organism Staphylococcus aureus (12). The anti-S. aureus AAC contains an antibody recognizing wall teichoic acid at the surface of S. aureus, a linker that can be cleaved by lysosomal cathepsins, and a rifamycin analog antibiotic (12). It was proposed that this AAC could eradicate extracellular S. aureus bacteria through phagocytosis followed by intracellular killing, and also eliminate preexisting intracellular reservoirs of bacteria. This AAC killed S. aureus intracellularly in macrophages, was efficacious in a mouse infection model (12), and has been tested in a phase I clinical trial (13). Thus, the AAC represents a potential novel therapeutic modality that could be used for infections that are difficult to treat with standard antibiotics.

In this study, we describe a proof of concept for an AAC molecule with potent activity against P. aeruginosa inside macrophages. The proposed mechanism of action of the AAC includes binding to the bacterial surface via the antibody portion, phagocytosis of AAC-bound bacteria, intracellular cleavage of the linker by cathepsins, and killing of the internalized bacteria by the AAC antibiotic released inside phagocytes. To what extent intracellular reservoirs contribute to human P. aeruginosa infections is currently unclear. The purpose of this anti-P. aeruginosa AAC is not to eradicate preexisting intracellular reservoirs, but rather to bring extracellular P. aeruginosa bacteria and the AAC molecule into the intracellular environment of phagocytic cells, to colocalize these bacteria with a high local concentration of free antibiotic and enable efficient killing. We hypothesized that this could enhance the anti-P. aeruginosa activity of an antibiotic which has moderate anti-P. aeruginosa activity as a free molecule. We observed that, after phagocytosis of AAC-bound P. aeruginosa bacteria, the AAC delivered a high intracellular concentration of the free antibiotic in macrophages, which was associated with efficient intracellular clearance of the P. aeruginosa bacteria. This study presents the second example of an active antibacterial AAC, which contains a different antibody and a different antibiotic and is directed to a different pathogen compared to the anti-S. aureus AAC. The current data show that a modestly active antibiotic can be potentiated by being concentrated in the intracellular environment of phagocytes, providing potential therapeutic applications for the treatment of recalcitrant infections.

## RESULTS

### Identification and characterization of anti-P. aeruginosa MAb 26F8.

**FIG 1:**
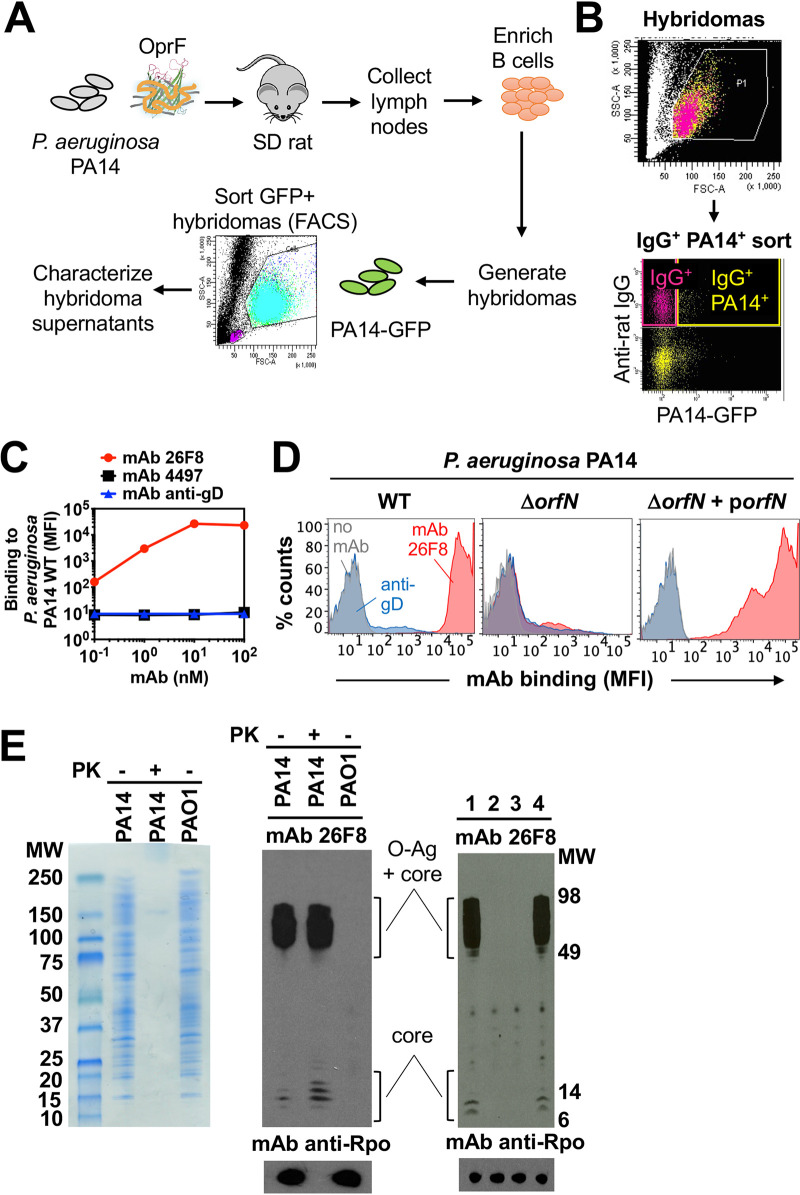
Generation and characterization of MAb 26F8 recognizing LPS O antigen on P. aeruginosa bacteria. (A) Schematic of the immunization and sorting procedure. Rats were immunized with P. aeruginosa PA14 bacteria and boosted with OprF beta-barrel protein reconstituted in amphipols. Rat lymph nodes were harvested, and purified B cells were fused with Sp2ab cells to generate hybridomas, which were subjected to fluorescence-activated cell sorting (FACS) based on binding to GFP-labeled P. aeruginosa PA14. Supernatants were purified, and clones were selected based on positive binding to whole P. aeruginosa bacteria, as determined by FACS. (B) FACS sorting profile of rat hybridomas to select P. aeruginosa-binding antibodies. Rat hybridoma cells were incubated with fluorescent anti-rat IgG antibodies and with P. aeruginosa PA14 bacteria expressing GFP and were sorted from the IgG^+^ PA14^+^ gate (yellow; upper right quadrant). (C) Intact P. aeruginosa PA14 WT bacteria were incubated with MAbs and fluorescently labeled anti-human secondary antibodies, followed by determination of the mean fluorescence intensity (MFI; arbitrary units) by flow cytometry. Rat MAb 26F8, engineered with human Fc (red circles), showed dose-dependent, high-intensity binding to P. aeruginosa. Binding was hardly detectable for isotype-matched anti-S. aureus MAb 4497 (black squares) or for MAb against cytomegalovirus gD (anti-gD; blue triangles). (D) MAb 26F8 demonstrated high-intensity binding to intact P. aeruginosa PA14 WT bacteria (left). MAb 26F8 did not show binding to PA14 Δ*orfN* bacteria, which lack LPS O antigen (middle). Binding of MAb 26F8 to PA14 Δ*orfN* was restored by complementation with pUCP19-*orfN* (p*orfN*) plasmid (right). Binding of MAb 26F8 to PA14 WT was represented by a shift in fluorescence of approximately 3 log compared to background fluorescence without antibody. Antibody binding was assessed by flow cytometry and was expressed as MFI. Red, MAb 26F8; blue, MAb anti-gD; gray, no MAb; MAbs were incubated at 10 nM. (E) (Left) Whole-cell lysates of P. aeruginosa PA14 WT or PAO1 WT were treated with or without proteinase K (PK), separated on SDS-PAGE gels, and stained with Coomassie. (Middle) Lysates of P. aeruginosa strains PA14 WT and PAO1 WT, treated with or without PK, were immunoblotted with MAb 26F8 (top) or, as a protein loading control, with MAb anti-RNA polymerase-α (Rpo) (bottom). (Right) Lysates of P. aeruginosa PA14 WT (lane 1), Δ*orfN* (lane 2), Δ*orfN* plus empty pUCP19 plasmid (lane 3), or Δ*orfN* plus pUCP19-*orfN* (lane 4) were immunoblotted with MAb 26F8 (top) or, as a loading control, with anti-Rpo MAb (bottom). MW, molecular weight marker.

To identify the monoclonal antibody (MAb) portion of the AAC molecule, we screened for antibodies capable of binding highly abundant P. aeruginosa surface antigens. A high antigen density leading to a high level of antibody binding is desirable, since the number of AAC molecules bound to the bacterial surface can be predicted to be proportional to the concentration of antibiotic molecules delivered intracellularly by the AAC. Sprague-Dawley rats were immunized by injections with live P. aeruginosa PA14 bacteria, followed by sequential boosters with recombinant P. aeruginosa OprF, which is known to be an abundant, conserved, and immunogenic P. aeruginosa outer membrane protein (14) (Fig. 1A). Hybridoma cells from immunized rats were sorted based on their capacity to bind intact fluorescent P. aeruginosa PA14 bacteria (Fig. 1A and B). This procedure yielded 476 sorted hybridomas; 102 of these were found to produce IgG, which were then subjected to purification. Of these purified IgG preparations, 5 showed binding to intact PA14 bacteria, as determined by flow cytometry. MAb 26F8 was selected, as it demonstrated the highest level of binding to intact PA14, represented by an approximately 3-log shift in fluorescence from background (Fig. 1C and D, left). As controls, isotype-matched anti-S. aureus MAb 4497 (12, 15) and anti-cytomegalovirus glycoprotein D (gD) (12) showed no binding to intact PA14 bacteria (Fig. 1C and D). MAb 26F8, as well as the other IgGs able to bind intact PA14 WT, showed a similar level of binding to intact PA14 Δ*oprF* mutant bacteria (Fig. S1). This indicated that the antigen recognized by MAb 26F8 was not OprF.

10.1128/mBio.00202-21.1 FIG S1 MAb 26F8 binds to intact P. aeruginosa PA14 WT and PA14 Δ*oprF* bacteria and not to P. aeruginosa PAO1 WT. MAb 26F8 (incubated with intact bacteria at 10 nM) showed similar strong binding to P. aeruginosa PA14 WT (serotype O11; left) and the PA14 Δ*oprF* mutant (middle). Binding of MAb 26F8 to P. aeruginosa PAO1 WT (serotype O5; right) was hardly detectable. Red, MAb 26F8; blue, isotype control MAb anti-gD; grey, no MAb. Antibody binding to whole P. aeruginosa bacteria was analyzed by flow cytometry, as described in the legend to Fig. 1C and D. Download FIG S1, TIF file, 0.4 MB. Copyright © 2021 Kajihara et al. 2021 Kajihara et al. https://creativecommons.org/licenses/by/4.0/ This content is distributed under the terms of the Creative Commons Attribution 4.0 International license.

To further characterize MAb 26F8, we sought to identify the antigen by using biochemical and genetic approaches. Treatment of PA14 lysates with proteinase K prior to Western blotting did not abolish antigen recognition by MAb 26F8, suggesting that its antigen is not proteinaceous (Fig. 1E, left and middle). Furthermore, Western blot analysis revealed that the antigen recognition by MAb 26F8 resembled a ladder pattern indicative of lipopolysaccharide (LPS) with O antigen (16) (Fig. 1E, middle). LPS O antigen of P. aeruginosa is known to display significant serotype variability (17, 18). We determined the LPS O-antigen serotypes of P. aeruginosa strains PA14 and PAO1 and observed them to be different (O10 and O5, respectively). Consistent with this, MAb 26F8 was unable to bind to lysates of strain PAO1 by Western blotting (Fig. 1E, middle) or intact PAO1 bacteria by flow cytometry (see Fig. S1 in the supplemental material). To genetically confirm the possibility that MAb 26F8 recognized LPS O antigen, we tested the reactivity of MAb 26F8 with a P. aeruginosa PA14 mutant strain lacking *orfN*. The *orfN* gene shares 66% identity with the P. aeruginosa PAO1 *wbpL* gene, which is required for LPS O-antigen assembly (19). MAb 26F8 did not bind the PA14 Δ*orfN* mutant strain, while binding was rescued by complementation with a plasmid expressing the *orfN* gene, as determined by flow cytometry using intact bacteria (Fig. 1D, middle and right) and by Western blotting using lysates (Fig. 1E, right). Together, these data support the conclusion that MAb 26F8 recognizes the LPS O antigen at the surface of P. aeruginosa PA14 bacteria.

### Generation of the anti-P. aeruginosa AAC 26F8-cBuCit-G2637.

**FIG 2:**
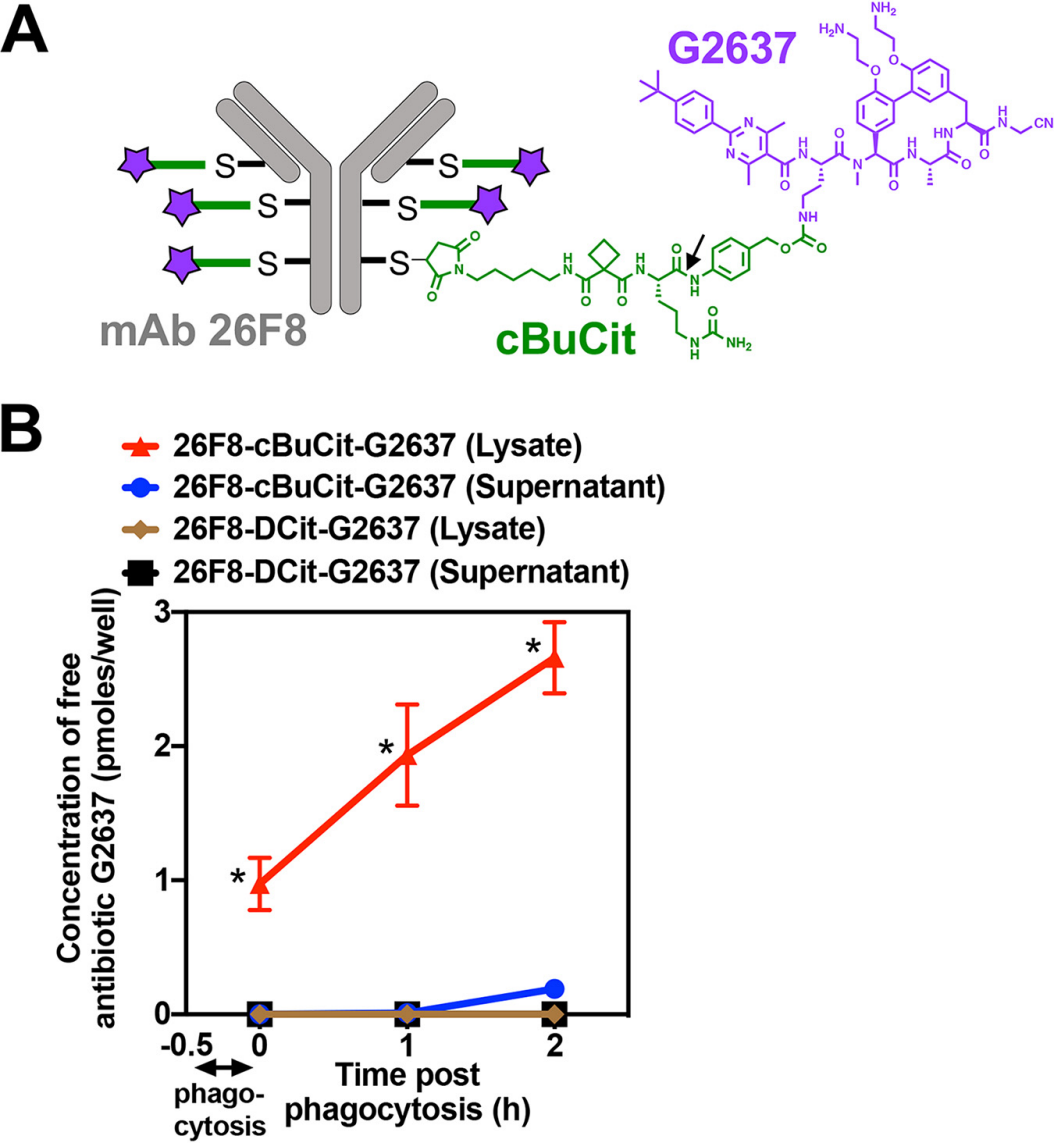
Composition of the AAC molecule and delivery of high AAC-released intracellular concentrations of free G2637 antibiotic into macrophages. (A) Schematic of the 26F8-cBuCit-G2637 AAC molecule, composed of MAb 26F8 (gray), which recognizes the LPS O antigen of P. aeruginosa PA14, cathepsin-cleavable cBuCit linker (green), and the arylomycin analog antibiotic G2637 (purple), with a drug-to-antibody ratio of 6. The arrow indicates the cathepsin cleavage site. (B) Determination of the amount of free AAC-released antibiotic G2637 in cell lysates or supernatants after phagocytosis of AAC-preincubated P. aeruginosa PA14 WT by LC-MS/MS analysis. When PA14 WT bacteria were incubated with 26F8-cBuCit-G2637, free G2637 was detected in lysates immediately after 30 min of phagocytosis by macrophages and removal of extracellular bacteria (0 h); this value increased during 2 h of subsequent incubation following phagocytosis, suggesting continued intracellular cleavage of the linker. Free G2637 was hardly detectable in the extracellular cell supernatant, indicating prolonged intracellular retention of the free AAC-released antibiotic. The AAC 26F8-DCit-G2637, which contains the noncleavable DCit linker, did not release detectable intracellular G2637. Value are averages ± SD for technical triplicates; asterisks indicate significant differences (*P* < 0.01) from values for 26F8-DCit-G2637.

After having identified MAb 26F8 recognizing intact P. aeruginosa PA14 bacteria, we re-engineered this MAb to contain 6 unpaired cysteines and conjugated it to a chemically synthesized linker-antibiotic molecule, in order to generate the AAC molecule. As the linker, we used cyclobutane-1,1-dicarboxamide citrulline (cBuCit), which is cleavable by lysosomal cathepsins and which enables release of unaltered drug molecules (20, 21). As the antibiotic, we used G2637, a close analog of the synthetic arylomycin derivative G0775, which targets the essential type I signal peptidase LepB of Gram-negative bacteria (22). As a free antibiotic, G2637 exhibited moderate anti-P. aeruginosa activity, as demonstrated by a MIC of 2.0 μM for wild-type (WT) PA14 in broth at pH 7 (Table S1). Free G2637 retained potency against PA14 at pH 5 (Table S1), which is relevant for the mechanism of action of the AAC, since the antibiotic is to be released in the acidic environment of the phagolysosome. Given that G2637 showed only moderate activity against P. aeruginosa as free antibiotic, we hypothesized that incorporation into an AAC molecule could enhance its local intracellular concentration to enable efficient killing of P. aeruginosa in the intracellular environment. Conjugation enabled the generation of the 26F8-cBuCit-G2637 AAC molecule (Fig. 2A), which exhibited an average drug-to-antibody ratio (DAR) of approximately 6, as confirmed by liquid chromatography-tandem mass spectrometry (LC-MS/MS) (Table 1).

**TABLE 1:** AAC, DAR, and requirement of linker cleavage for anti-P. aeruginosa activity

Molecule	Description	DAR^*a*^	G2637 MIC (μM) for P. aeruginosa PA14 WT at cathepsin B concn (nM)^*b*^	
			0	500
G2637	Free antibiotic	NA	2.7 ± 1.2	3.3 ± 1.2
26F8-cBuCit-G2637	Cleavable AAC with anti-P. aeruginosa MAb 26F8	5.7	>16	4.0 ± 0.0
26F8-DCit-G2637	Noncleavable AAC with anti-P. aeruginosa MAb 26F8	5.7	>16	>16
4497-cBuCit-G2637	Cleavable AAC with anti-S. aureus MAb 4497	6.0	>16	4.0 ± 0.0

a For each AAC molecule, the DAR was determined by LC-MS/MS analysis (Fig. S2). NA, not applicable.

b Free G2637 antibiotic and AAC molecules were incubated with medium containing no cathepsin B (0 nM) or 500 nM purified cathepsin B for 1 h at 37°C. The activity of the cleavage products against P. aeruginosa PA14 WT was determined in a standard MIC assay and is expressed as concentration of G2637 antibiotic payload; data are averages ± SD for biological triplicates.

10.1128/mBio.00202-21.6 TABLE S1 MICs of G2637 for Gram-negative bacteria, as determined in MHB at pH 7 or pH 5. MICs were determined using the standard protocol from the Clinical and Laboratory Standards Institute. Values are averages ± SD for biological triplicates. Nd, not determined. Download Table S1, DOCX file, 0.02 MB. Copyright © 2021 Kajihara et al. 2021 Kajihara et al. https://creativecommons.org/licenses/by/4.0/ This content is distributed under the terms of the Creative Commons Attribution 4.0 International license.

10.1128/mBio.00202-21.2 FIG S2 LC-MS/MS analysis to determine the drug-to-antibody ratio (DAR) of 26F8-DCit-G2637 AAC. (A) LC-MS/MS analysis of the conjugate shows the TCI and UV trace. (B) Deconvoluted spectra of the LC-MS analysis showing the light chain (LC) and light chain plus 1 G2637 antibiotic, as well as heavy chain (HC) plus 1, 2, or 3 G2637 antibiotic species. The DAR was calculated using the abundance of the ions present in LC-MS/MS deconvoluted results (DAR values are shown in Table 1). Details of the procedure are given in Materials and Methods. Download FIG S2, TIF file, 0.4 MB. Copyright © 2021 Kajihara et al. 2021 Kajihara et al. https://creativecommons.org/licenses/by/4.0/ This content is distributed under the terms of the Creative Commons Attribution 4.0 International license.

### Requirement of linker cleavage for anti-P. aeruginosa activity of AAC molecules.

Next, to investigate whether antibacterial activity of the 26F8-cBuCit-G2637 AAC required cleavage and release of free G2637, we incubated P. aeruginosa PA14 WT bacteria with AAC molecules that had been treated with purified cathepsin B. Untreated 26F8-cBuCit-G2637 AAC did not inhibit growth of P. aeruginosa in broth (Table 1). Cathepsin B treatment of either 26F8-cBuCit-G2637 AAC or 4497-cBuCit-G2637 AAC, which contains the anti-S. aureus MAb 4497, resulted in growth inhibition of P. aeruginosa, the MIC of AAC-released G2637 for PA14 WT in broth being similar to that of free G2637 antibiotic (Table 1). In contrast, cathepsin B treatment of 26F8-DCit-G2637 AAC, which contains the noncleavable DCit linker, did not lead to growth inhibition of P. aeruginosa (Table 1). These data demonstrated that linker cleavage is required for anti-P. aeruginosa activity of the AAC and that the G2637 antibiotic retained its antibacterial activity after being released from the AAC.

### Anti-P. aeruginosa AAC delivers a high intracellular concentration of free antibiotic.

Since an important component of the proposed mechanism of action of AAC involved the intracellular accumulation of released antibiotic, we measured the intracellular concentration of free G2637 antibiotic that could be delivered by AAC cleavage inside macrophages. P. aeruginosa PA14 WT bacteria were incubated with 26F8-cBuCit-G2637 AAC and added to RAW264.7 macrophages, followed by removal of extracellular bacteria and quantification of levels of free G2637 antibiotic in macrophage lysates by mass spectrometry. A significant amount of free G2637 was detected in the macrophage lysates immediately after phagocytosis, which further increased during prolonged incubation (Fig. 2B). These data indicate that cleavage of the AAC linker had rapidly started during phagocytosis of bacteria and continued thereafter. No extracellular free G2637 antibiotic was detectable in the cell supernatant (Fig. 2B), indicating that intracellular free G2637 remained well retained inside the macrophages and did not diffuse out of the cells. In addition, no intracellular free G2637 antibiotic was detected when the noncleavable AAC 26F8-DCit-G2637 (Fig. 2B) was used, confirming the requirement of linker cleavage for the intracellular release of free antibiotic.

To estimate intracellular molar concentrations of G2637 in macrophages, we then converted the lysate values, which were expressed in picomoles per well (Fig. 2B), by using estimated cell volumes. These calculations revealed that the 26F8-cBuCit-G2637 AAC delivered an estimated intracellular concentration of free G2637 antibiotic of 5.8 ± 1.2 μM (mean ± standard deviation [SD]) in macrophages immediately after 30 min of phagocytosis, which subsequently increased to 11.6 ± 2.3 μM and 16.0 ± 1.6 μM during 1 h and 2 h of incubation postphagocytosis, respectively. Since 100 nM extracellular AAC had been added at the start of the experiment, which given its DAR of 6 corresponded to a concentration of G2637 antibiotic of 600 nM, these data indicated that the AAC had locally increased the concentration of G2637 by approximately 26-fold within the intracellular environment. The estimated AAC-released intracellular free G2637 concentrations were well above the MIC of G2637 for P. aeruginosa PA14 WT (Table S1). Together, these data demonstrated that the 26F8-cBuCit-G2637 AAC molecule delivered and maintained a high concentration of free antibiotic inside macrophages upon intracellular cleavage.

### 26F8-cBuCit-G2637 AAC mediates potent intracellular killing of P. aeruginosa in macrophages.

**FIG 3:**
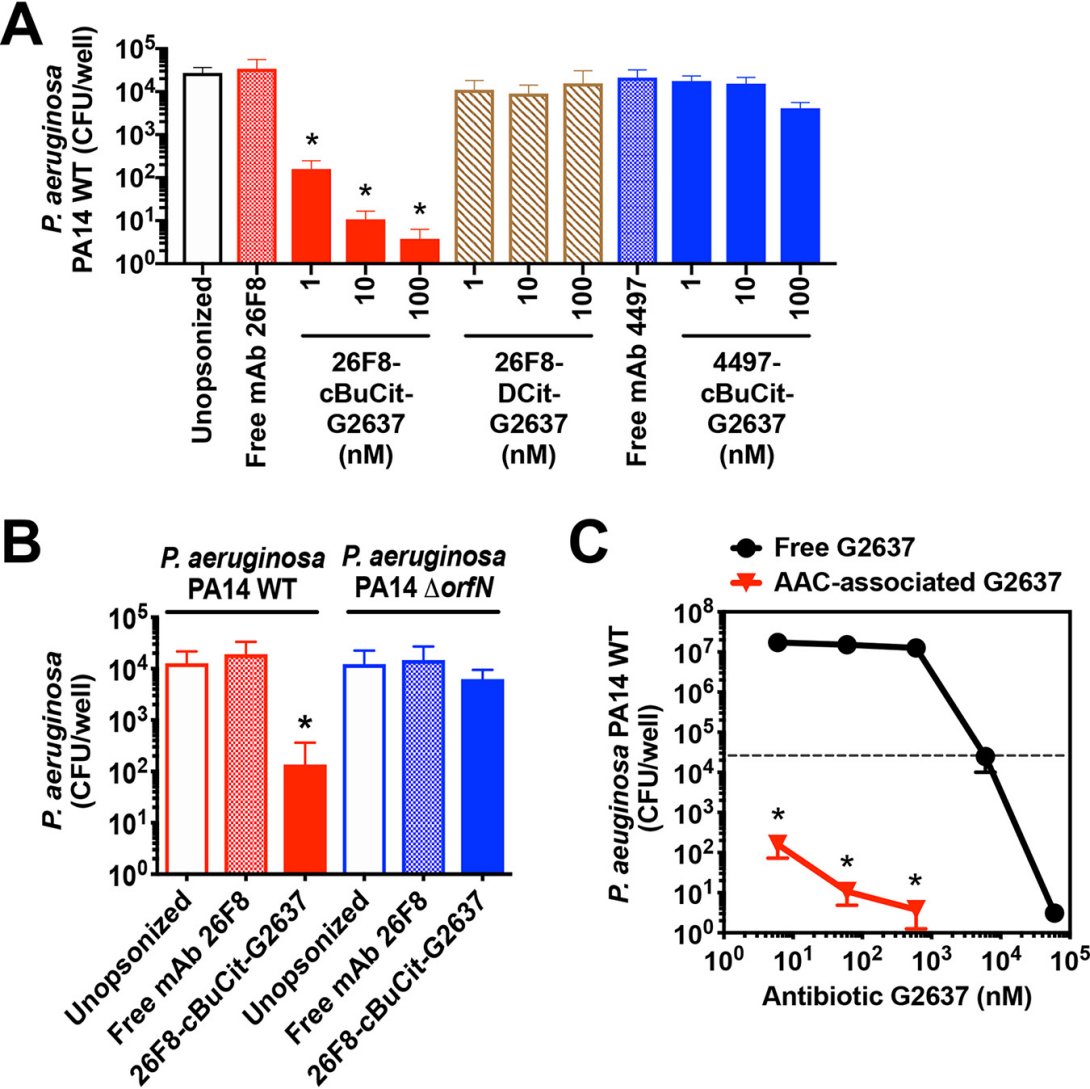
26F8-cBuCit-G2637 AAC induces potent intracellular killing of P. aeruginosa in macrophages. To determine anti-P. aeruginosa potency of AAC, P. aeruginosa bacteria were preincubated with AAC (1, 10, or 100 nM) or with free MAb (100 nM) and added to macrophages to induce phagocytosis. After addition of gentamicin to remove extracellular bacteria, macrophages were subsequently incubated for an additional 6 h at 37°C to enable bacterial killing, followed by macrophage lysis and CFU enumeration. (A) The 26F8-cBuCit-G2637 AAC (solid red bars) induced potent and dose-dependent intracellular killing of P. aeruginosa PA14 WT bacteria. In contrast, the viability of intracellular P. aeruginosa was hardly affected when bacteria were left unopsonized (empty bar) or preincubated with noncleavable 26F8-DCit-G2637 DAR6 AAC (hatched bars) or 4497-cBuCit-G2637 DAR6, which contains anti-S. aureus MAb 4497 (solid blue bars), or with free anti-P. aeruginosa MAb 26F8 (cross-hatched red bar) or free MAb 4497 (cross-hatched blue bar). (B) The 26F8-cBuCit-G2637 AAC promoted intracellular killing of P. aeruginosa PA14 WT (solid red bar) but not of the P. aeruginosa PA14 Δ*orfN* mutant (solid blue bar), which lacks the LPS O-antigen required for binding of MAb 26F8 (Fig. 1D and E). (A and B) Data are averages and SD for biological triplicates; asterisks indicate statistical significance (*P* < 0.05) compared to the CFU value of each condition immediately after phagocytosis (values are plotted in Fig. S3). (C) PA14 WT bacteria were preincubated with 26F8-cBuCit-G2637 AAC, followed by determination of killing 6 h postphagocytosis as for panel A (red triangles), or were incubated with free G2637 antibiotic for 6 h in the same medium without macrophages (black circles); viability of bacteria is expressed as CFU per well. 26F8-cBuCit-G2637-associated G2637 antibiotic required a molar concentration to kill P. aeruginosa PA14 WT approximately 2 orders of magnitude lower than that of free extracellular antibiotic G2637. The dashed line indicates input bacterial concentration at the start of the assay before the 6 h of incubation, which was in the same range for both conditions (approximately 2 × 10^4^ to 5 × 10^4^/ml). Value are averages ± SD for biological triplicates; asterisks indicate significant differences (*P* < 0.01) compared to free G2637 for each concentration.

We next determined whether the high intracellular levels of G2637 antibiotic after release from 26F8-cBuCit-G2637 AAC would enable intracellular killing of P. aeruginosa. To test this, P. aeruginosa bacteria were incubated with 26F8-cBuCit-G2637 AAC and added to macrophages to induce phagocytosis. Extracellular bacteria were removed, and viable CFU were enumerated 6 h postphagocytosis. The 26F8-cBuCit-G2637 AAC molecule induced dose-dependent intracellular killing of P. aeruginosa PA14 WT (Fig. 3A). During the 6 h of incubation, the numbers of CFU were reduced by approximately 2 to 3 log, compared with the initial CFU numbers recovered immediately after phagocytosis (Fig. S3).

10.1128/mBio.00202-21.3 FIG S3 Numbers of macrophage-associated CFU of P. aeruginosa PA14 WT immediately after 30 min of phagocytosis. (A and B) The experimental conditions correspond to the experiments that are described in the legend to Fig. 3A and B, respectively. Indicated at the *x* scale are the various molecules used for preopsonization. No significant difference in the extent of phagocytosis was observed between the various conditions. Download FIG S3, TIF file, 0.5 MB. Copyright © 2021 Kajihara et al. 2021 Kajihara et al. https://creativecommons.org/licenses/by/4.0/ This content is distributed under the terms of the Creative Commons Attribution 4.0 International license.

No loss in intracellular CFU was observed when the noncleavable AAC 26F8-DCit-G2637 was used (Fig. 3A), confirming that linker cleavage is essential for activity. Furthermore, no killing was detected when unopsonized PA14 WT bacteria or free MAb 26F8 were used (Fig. 3A), demonstrating that the complete AAC molecule is required for intracellular killing.

No intracellular clearance was seen when the AAC was unable to bind to the bacteria, i.e., when PA14 WT was incubated with 4497-cBuCit-G2637 AAC, which contains the anti-S. aureus MAb 4497 (Fig. 3A), or when PA14 Δ*orfN* mutant bacteria were incubated with 26F8-cBuCit-G2637 AAC (Fig. 3B) prior to phagocytosis. The numbers of PA14 WT bacteria associated with macrophages immediately after phagocytosis did not significantly differ for these controls, i.e., free MAb, 26F8-DCit-G2637, and 4497-cBuCit-G2637, or for the PA14 Δ*orfN* mutant (Fig. S3). Therefore, the absence of intracellular killing for these controls could not be explained by differences in efficiency of phagocytosis. In addition, we observed that preincubation of P. aeruginosa PA14 WT with free MAb 26F8 or AAC did not enhance the number of macrophage-associated bacteria, compared to unopsonized bacteria (Fig. S3). Thus, the role of this MAb in AAC activity is likely not related to promoting phagocytosis, but rather to binding the bacterial surface and colocalizing the bacteria with the cleavable linker-antibiotic in the intracellular environment.

The molar concentration of AAC-associated G2637 antibiotic (i.e., input concentration in the intracellular killing assay) that resulted in complete killing of PA14 WT in macrophages was approximately 2 orders of magnitude lower than the concentration of free G2637 antibiotic required to kill extracellular PA14 WT (Fig. 3C). This indicates that the AAC was more potent than extracellular free antibiotic on a molar basis. In addition, this supports the hypothesis that the AAC would enhance the activity of a moderately active antibiotic by enhancing the local concentration inside macrophages. While the intracellular concentration of AAC-released G2637 antibiotic had reached high levels (approximately 16 μM at 2 h after phagocytosis, as noted above), which were close to the concentration of free antibiotic able to fully kill extracellular PA14 (Fig. 3C), it cannot be excluded that macrophage antibacterial mechanisms also contributed to the efficient intracellular killing.

Cumulatively, the current data demonstrate that the 26F8-cBuCit-G2637 AAC delivers a high intracellular concentration of free antibiotic, enabling efficient intracellular killing of P. aeruginosa, and that AAC binding to the bacteria and linker cleavage are essential for its mechanism of action.

## DISCUSSION

The data presented in this study demonstrate that the anti-P. aeruginosa AAC 26F8-cBuCit-G2637 delivers a high intracellular concentration of the arylomycin analog G2637 upon phagocytosis, resulting in potent clearance of P. aeruginosa in macrophages. This AAC molecule was significantly more potent than extracellular free antibiotic on a molar basis. The proposed mechanism of action was supported by the following observations. First, binding of the 26F8-cBuCit-G2637 AAC to P. aeruginosa bacteria was required for activity. Second, cleavage of the linker was required for AAC activity. Third, high intracellular concentrations of active G2637 antibiotic were released upon AAC cleavage, exceeding the MIC of free antibiotic for P. aeruginosa.

In generating the proof-of-concept 26F8-cBuCit-G2637 AAC molecule, we also devised an efficient method of screening for antibodies that recognize bacterial surface-exposed antigens. Binding to a highly expressed antigen was expected to be a critical feature of an effective AAC, since the number of intracellularly delivered antibiotic molecules would be proportional to the number of AAC molecules bound per bacterial cell.

Although no OprF-specific antibodies able to bind whole WT P. aeruginosa PA14 bacteria were identified, the identification of MAb 26F8, which binds to the highly expressed LPS O antigen, validates this screening strategy. The lack of identified anti-OprF antibodies may be related to differences between recombinant and native OprF protein or to reduced accessibility of OprF on bacterial cells to antibodies. Indeed, previous studies using mutant Escherichia coli strains with truncated LPS O antigen indicated that LPS O antigen excludes, at least partially, binding of antibodies to outer membrane proteins such as porins, LptD, and BamA and that antibodies able to access these proteins in WT bacteria are rare (23–25). Therefore, to successfully find anti-OprF antibodies with WT binding capacity, a larger number of hybridomas would likely need to be screened. In addition to the above-mentioned surface antigen density, other critical features supporting the AAC activity are the retained anti-P. aeruginosa potency of free antibiotic G2637 at low pH, reflecting the phagolysosomal environment, and the prolonged intracellular retention of AAC-released G2637 inside macrophages.

Building upon our previously described anti-S. aureus AAC (12), the current study indicates that the AAC platform may be applied to other bacterial pathogens. The 26F8-cBuCit-G2637 DAR6 AAC showed potent anti-P. aeruginosa activity, despite the fact that the G2637 antibiotic had only modest anti-P. aeruginosa activity as free antibiotic, i.e., in a single-digit micromolar range. In comparison, the rifamycin analog antibiotic, which was used in the previously described anti-S. aureus DAR2 AAC, exhibited antibacterial activity as free drug in the single-digit nanomolar range (12). Thus, the current format of anti-bacterial AAC may be applicable to a broader spectrum of antibiotics that do not need intrinsic nanomolar activity as free drug. In this context, it is noteworthy that for antitumor ADC molecules to be active, the choice of cytotoxic payloads is more restricted, i.e., requiring activity in the picomolar range (11). Our data support the hypothesis that antibiotics with only modest antibacterial activity can be significantly potentiated by incorporation into an AAC molecule.

To further develop an anti-P. aeruginosa AAC as a clinical candidate, a MAb with broader reactivity to predominant clinical P. aeruginosa isolates would be required, given that the LPS O antigen, which is recognized by MAb 26F8, displays significant serotype variability (20). Additional flow cytometry counterscreens could be incorporated using bacterial cells expressing different LPS O-antigen serotypes or O-antigen-deficient PA14, in order to identify broadly reactive clinical candidate antibodies. In addition, future experiments will be needed to test whether anti-P. aeruginosa AAC molecules are efficacious in animal models of infection. Using a previously reported mouse model of acute P. aeruginosa pneumonia (26), our initial *in vivo* experiments did not show therapeutic efficacy of systemically administered 26F8-cBuCit-G2637 AAC against PA14. We speculate that this may be explained by several possibilities, such as reduced antigen expression or insufficient AAC exposure at the site of infection in the lung. Also, we cannot exclude the possibility that in this mouse model, phagocytosis at the site of infection was relatively inefficient, and that including a MAb with stronger opsonic activity in the AAC molecule could potentially promote *in vivo* efficacy.

The limited clinical success of antibiotic treatment of P. aeruginosa infections, leading to high mortality rates, can be related to a number of factors, including intrinsic or acquired antibiotic resistance, formation of biofilms, bacterial genetic adaptability, and weakened host immunity (27). Raising antibiotic concentrations at the site of infection could potentially address some of these problems; however, since systemic antibiotics are often dose limited due to adverse effects, alternative strategies are needed. The AAC molecule presented here is specifically designed to colocalize P. aeruginosa with high antibiotic concentrations in the intracellular environment of phagocytic cells, providing additional therapeutic options for moderately active antibiotics. Further understanding of the behavior of the anti-P. aeruginosa AAC at the site of infection in the host environment will be needed to enable optimization for *in vivo* use.

In conclusion, this proof-of-concept study suggests that the AAC strategy has the potential to enhance clearance of P. aeruginosa infections, compared to what can be achieved by antibiotic alone, by inducing high intracellular antibiotic concentrations and intracellular bacterial elimination. The AAC strategy could have therapeutic applicability to antibiotics with limited clinical utility due to moderate antibacterial activity or unfavorable pharmacokinetic or systemic toxicity profiles.

## MATERIALS AND METHODS

### Bacterial strains.

P. aeruginosa strain PA14 was cultured at 37°C in Luria-Bertani broth (LB), Mueller-Hinton broth (MHB), or on Mueller-Hinton agar (MHA) plates. Strains, plasmids, primers, DNA sequences, and antibodies are specified in Table S2.

10.1128/mBio.00202-21.7 TABLE S2 Strains, plasmids, antibodies, primers, and DNA sequences used in this study. Download Table S2, DOCX file, 0.03 MB. Copyright © 2021 Kajihara et al. 2021 Kajihara et al. https://creativecommons.org/licenses/by/4.0/ This content is distributed under the terms of the Creative Commons Attribution 4.0 International license.

Allelic replacement strains were constructed by using an unmarked, non‐polar deletion strategy (28, 29). To delete the LPS O antigen in P. aeruginosa PA14, flanking regions of *orfN* were amplified using gene-specific primer sets. The PCR products were cloned into the sucrose-based suicide vector pEX100T (ATCC, Manassas, VA) using the Gibson Assembly cloning kit (New England BioLabs, Ipswich, MA). The plasmid was verified by sequence analysis (ELIM Biopharmaceuticals, Hayward, CA) and transferred into the P. aeruginosa PA14 Δ*R2* pyocin mutant strain by conjugation via E. coli S17. The P. aeruginosa PA14 Δ*R2* pyocin strain was used to enable efficient knockout of the *orfN* gene (30). Single recombination mutants were selected on LB agar containing 100 μg/ml carbenicillin and 25 μg/ml Irgasan. Double-recombination mutants were selected on LB plates without NaCl, containing 10% sucrose, and confirmed by PCR and sequence analysis. To complement P. aeruginosa PA14 Δ*orfN* with the *orfN* gene, *orfN* was amplified from P. aeruginosa PA14 genomic DNA and cloned into the pUCP19 plasmid (ATCC) using the In-Fusion HD EcoDry cloning kit (TaKaRa Bio, Mountain View, CA). The resultant plasmid was verified by sequence analysis (ELIM Biopharmaceuticals, Hayward, CA).

To delete the *oprF* gene in PA14, the flanking regions of *oprF* were cloned into the sucrose-based suicide vector pEX100T (ATCC, Manassas, VA) using the In-Fusion HD EcoDry cloning kit (TaKaRa Bio). The plasmid was transferred into PA14 WT by conjugation via E. coli S17, and recombination steps were carried out as described above for the PA14 Δ*orfN* mutant.

LPS O-antigen serotypes of P. aeruginosa PA14 and PAO1 were determined by using the P. aeruginosa antiserum serotyping kit (Denka Seiken, Tokyo, Japan).

To generate green fluorescent protein (GFP)-expressing P. aeruginosa PA14, enhanced GFP (EGFP) (DASHERgfp; DNA2.0) was cloned into the broad-host-range vector pBHR1 (MoBiTec) under the control of the chloramphenicol promoter, using the In-Fusion HD EcoDry cloning kit (TaKaRa Bio). The kanamycin resistance gene was replaced with the gene conferring gentamicin resistance for efficient selection of P. aeruginosa. After transformation of P. aeruginosa PA14 WT with pBHR1-GFP by electroporation, GFP-expressing PA14 colonies were cultured in LB containing 30 μg/ml gentamicin, and GFP expression was confirmed by flow cytometry.

Experiments to determine MICs were performed according to the standard protocol from the Clinical and Laboratory Standards Institute.

### Protein expression and purification.

Sequences were obtained from Uniprot.org. DNA encoding the signal sequence of Escherichia coli OmpA (residues M1 to A21), followed by an N-terminal His8 tag, and the beta-barrel domain of P. aeruginosa OprF (residues Q25 to F184) was synthesized and subcloned into a modified version of the pET52b expression vector under the control of the T7 *lac* promoter.

BL21(DE3) competent cells (New England Biolabs) were transformed with the expression plasmid according to the manufacturer’s instructions. A single colony was inoculated into 50 ml LB medium containing 50 μg/ml carbenicillin and grown overnight at 37°C with shaking at 200 rpm. The next day, the culture was diluted 1:100 into two 2-liter portions of TB autoinduction medium with trace metals and 50 μg/ml carbenicillin in a 2.5-liter Ultra Yield flask (Thomson Instrument Company, Oceanside, CA) and grown at 17°C for 48 h, as previously described (31). Cells were harvested by centrifugation at 8,000 rpm for 20 min, and the pellet was resuspended in 300 ml of lysis buffer (50 mM Tris-HCl [pH 8.0], 200 mM NaCl, EDTA-free cOmplete protease inhibitor [Roche], 10 mg/liter DNase I [Sigma-Aldrich, St. Louis, MO], 200 mg/liter lysozyme [Sigma-Aldrich]). Cells were lysed with four passes over a microfluidizer, and 12 g of beta-octyl glucoside (Anatrace, Maumee, OH) was added to the lysate, followed by rotation at 4°C for 4 h to solubilize the membrane fraction. Lysates were clarified by centrifugation at 40,000 rpm for 30 min at 4°C, filtered through a 0.22-μm filter, and loaded onto a 3-ml nickel-nitrilotriacetic acid (Ni-NTA) (Cytiva) column using an AKTA Pure system (Cytiva, Marlborough, MA). The column was washed with 30 column volumes of buffer A (50 mM Tris-HCl [pH 8.0], 200 mM NaCl, 1.5% [wt/vol] beta-octyl glucoside, 10 mM imidazole), and protein was eluted with buffer B (50 mM Tris-HCl [pH 8.0], 200 mM NaCl, 1.5% beta-octyl glucoside, 400 mM imidazole) into a sample loop and directly injected over a HiLoad 16/600 Superdex 75 prep-grade column (Cytiva) with 50 mM Tris-HCl (pH 8.0), 200 mM NaCl, 1.5% beta-octyl glucoside as the mobile phase.

Fractions were analyzed by SDS-PAGE and pooled, and OprF at 1.2 mg/ml was reconstituted in 5 mg/ml amphipol A8-35 (Anatrace) with incubation at 4°C overnight. The next day, detergent was removed by incubation with Bio-Beads (Bio-Rad, Hercules, CA) at 4°C for 2 h, according to the manufacturer’s instructions. Excess amphipol was removed by purification over a HiLoad 16/600 Superdex 75 prep-grade column (Cytiva) with 50 mM Tris-HCl (pH 8.0), 200 mM NaCl as the mobile phase.

### Generation of antibodies.

To generate anti-P. aeruginosa hybridoma antibodies, Sprague-Dawley rats (Charles River, Hollister, CA) were immunized three times by two intradermal injections of 10^7^ CFU of live P. aeruginosa PA14 at 2-week intervals, followed by two subcutaneous (s.c.) and intraperitoneal (i.p.) injections, each with 25 μg of OprF beta-barrel protein in amphipols, with a 2-week interval. Preliminary studies demonstrated that two doses of PA14 of 10^7^ CFU resulted in significant serum reactivity against whole P. aeruginosa bacteria (Fig. S4). Multiple lymph nodes were harvested 3 days after the last immunization. IgM-negative B cells were purified using magnetic separation (Miltenyi Biotec, San Diego, CA) and fused with Sp2ab mouse myeloma cells (Abeome, Athens, GA) using electrofusion (Harvard Apparatus, Holliston, MA). Fused cells were cultured at 37°C and 7% CO_2_ in Clonacell-HY medium C (Stemcell Technologies, Vancouver, BC, Canada). Next day, the cells were centrifuged, resuspended in Clonacell-HY medium E (Stemcell Technologies) supplemented with hypoxanthine-aminopterin-thymidine (HAT) (Sigma-Aldrich), and cultured in 12-well plates at 37°C and 7% CO_2_. Four days later, single hybridoma cells showing reactivity with both anti-rat IgG-allophycocyanin (APC) (Jackson ImmunoResearch, West Grove, PA) and intact P. aeruginosa PA14-GFP organisms were sorted using a FACSAria III sorter (BD, Franklin Lakes, NJ) and collected in 96-well plates containing medium E (Stemcell Technologies). Seven days later, supernatants were tested for IgG expression by enzyme-linked immunosorbent assay (ELISA) using anti-rat IgG; IgG-containing supernatants were tested for reactivity with intact P. aeruginosa PA14 organisms by flow cytometry. Cell lines demonstrating binding to P. aeruginosa PA14 organisms were expanded, and supernatants were harvested and purified using protein G Sepharose GammaBind Plus (GE Healthcare, Pittsburgh, PA). Hybridoma MAb 26F8 was selected based on its high-intensity binding to P. aeruginosa PA14.

10.1128/mBio.00202-21.4 FIG S4 Sera from immunized rats showing IgG binding to wild-type P. aeruginosa PA14 WT bacteria. Rats were immunized by 2 or 3 intradermal injections of live P. aeruginosa PA14 WT (10^5^, 10^6^, 10^7^, or 10^8^ CFU per dose). Serum was incubated with intact P. aeruginosa PA14 WT bacteria, followed by fluorescently labeled anti-rat IgG antibody and analysis of mean fluorescence intensity (MFI) by flow cytometry, as a measure for IgG binding. Rats that received 10^7^ CFU per injection were selected for hybridoma isolation. Download FIG S4, TIF file, 0.5 MB. Copyright © 2021 Kajihara et al. 2021 Kajihara et al. https://creativecommons.org/licenses/by/4.0/ This content is distributed under the terms of the Creative Commons Attribution 4.0 International license.

The DNA sequence of rat hybridoma 26F8 was determined by 5′ RACE (rapid amplification of cDNA ends) (SMARTscribe; TaKaRa Bio USA, Mountain View, CA) using purified total RNA (Qiagen, Germantown, MD) and gene-specific rat constant-region oligonucleotide primers. The cDNA encoding the 26F8 rat heavy- and light-chain sequences were amplified by PCR (TaKaRa Bio USA). DNA sequences of the antibody variable heavy chain and light chain were determined by Sanger sequencing. To create a rat/human 26F8 chimeric antibody, a second PCR amplification was performed with oligonucleotide adapters that were designed to enable ligation of the variable heavy-chain region and the variable light-chain region with mammalian cell expression vectors encoding a human heavy-chain IgG1 constant region and a human light-chain kappa constant region, respectively. An additional variant of the 26F8 chimeric antibody was made with the cysteine substitutions Leu174Cys and Tyr373Cys in the constant-region heavy chain and the substitution Lys149Cys in the constant light chain to produce THIOMABs™ (i.e., antibodies containing engineered reactive cysteine residues), enabling conjugation of multiple linker-antibiotic molecules per antibody molecule (see below). All substitutions were inserted using Kunkel mutagenesis (32). The substitutions were verified by DNA sequencing.

Generation of the human MAb 4497 recognizing the β-GlcNAc epitope of Staphylococcus aureus wall teichoic acid was described previously (12, 15). MAb 4497 was engineered with cysteines to produce THIOMABs™ following a procedure similar to that for MAb 26F8.

### Flow cytometry analysis of antibody binding to whole P. aeruginosa bacteria.

P. aeruginosa PA14 bacteria were grown to log phase in LB, washed, and resuspended at approximately 3.0 × 10^8^ cells/ml of Hanks’ balanced salt solution supplemented with 10 mM HEPES and 0.1% bovine serum albumin (BSA), pH 7 (HB). Primary antibodies were added at various concentrations and incubated for 1 h at room temperature (RT). Bacteria were washed and incubated with Alexa Fluor 647-conjugated donkey anti-human IgG (heavy plus light chain [H+L]) F(ab′)_2_ fragments for 1 h at RT. Bacteria were washed and fixed in phosphate-buffered saline (PBS) containing 2% paraformaldehyde at 4°C overnight, followed by flow cytometry analysis using FACSymphony and FACSDiva software (Becton, Dickinson, Franklin Lakes, NJ). Mean fluorescence intensity (MFI) was used as a measure for the level of antibody binding to whole P. aeruginosa bacteria.

### Western blot analysis of antibody binding to P. aeruginosa lysates.

P. aeruginosa bacteria were grown to log phase (with an optical density at 600 nm [OD_600_] of approximately 0.5) in LB, and 1 ml of culture was pelleted and resuspended in 0.1 ml of Laemmli sample buffer (Bio-Rad) with 4% beta-mercaptoethanol (Bio-Rad) and boiled for 10 min. The resulting lysates were loaded (2.5 to 5 μl per lane) and run on a 4-to-12% bis-Tris NuPAGE gel (Thermo Fisher Scientific, Waltham, MA) with 1× morpholineethanesulfonic acid (MES)/SDS running buffer (Thermo Fisher). In some instances, proteinase K (2.5-mg/ml final concentration; New England Biolabs) was added to the lysates and incubated for 1 h at 60°C prior to loading onto the gel. Proteins were transferred to nitrocellulose membranes (Thermo Fisher), which were incubated for 1 h at RT in blocking buffer (5% nonfat milk in 0.05% Tween 20, 50 mM Tris-HCl, 150 mM NaCl; pH 7.5), washed, and incubated for 1 h at RT with MAb 26F8 (1 μg/ml) or with mouse MAb 4RA2 anti-RNA polymerase-α (0.5 μg/ml; BioLegend, San Diego, CA), as a loading control, in blocking buffer. Next, blots were incubated for 1 h at RT with horseradish peroxidase-conjugated donkey anti-human IgG (H+L) or anti-mouse IgG (H+L) Affinipure F(ab′)_2_ fragment (Jackson ImmunoResearch) in blocking buffer and developed using ECL Prime Western blotting detection reagent (GE Healthcare).

### Conjugation of antibodies to linker-antibiotic molecules to generate AAC molecules.

Construction and production of the THIOMABs™ were done as reported previously (33). The synthesis of the linker-antibiotic molecule cBuCit-G2637, consisting of the cleavable cBuCit linker (20) and the P. aeruginosa PA14-active arylomycin analog G2637 (Table S1) targeting the Gram-negative signal peptidase LepB (22), is described in the supplemental methods (Text S1). The THIOMABs™ 26F8 and 4497 were conjugated to linker-antibiotic cBuCit-G2637 (schematic in Fig. 2A) as described previously (33). Briefly, the antibodies were reduced in the presence of a 100-fold molar excess of dithiothreitol (DTT) (Calbiochem, Billerica, MA) overnight. The reducing agent and the cysteine and glutathione blocks were removed using HiTrap SP-HP column (GE Healthcare). The antibodies were reoxidized in the presence of a 15-fold molar excess of dehydroascorbic acid (dhAA) (MP Biomedical) for 2.5 h. The formation of interchain disulfide bonds was monitored by LC/MS. An 8- or 18-fold molar excess of linker drug over protein was incubated in the presence of 15% dimethylformamide (DMF) with the activated THIOMABs™ for 3 or 18 h. The antibody-antibiotic conjugates were purified using Zeba desalting columns to remove excess linker drug. If aggregation of more than 5% was observed by analytical size exclusion chromatography (SEC), the conjugates were further purified using a Hi Load Superdex 200 pg 16/600 column (GE Healthcare) with 20 mM histidine-acetate, 150 mM NaCl (pH 5.5) with 15% isopropanol (IPA) as running buffer. The number of conjugated linker-antibiotic molecules per THIOMAB™ was quantified by LC/MS analysis. Purity was also assessed by size exclusion chromatography.

10.1128/mBio.00202-21.8 TEXT S1 Supplemental method. Synthesis of linker-antibiotic molecule cBuCit-G2637. Download Text S1, DOCX file, 0.6 MB. Copyright © 2021 Kajihara et al. 2021 Kajihara et al. https://creativecommons.org/licenses/by/4.0/ This content is distributed under the terms of the Creative Commons Attribution 4.0 International license.

### Determination of efficiency of AAC linker cleavage.

To determine the efficiency of AAC linker cleavage, 26 μM AAC was incubated with 500 nM purified cathepsin B (from bovine spleen; Calbiochem) in cathepsin cleavage buffer (20 mM sodium acetate, 1 mM EDTA, 5 mM l-cysteine; pH 5.0) for 1 h at 37°C. The reaction was stopped by addition of 9 volumes of MHB. After culture for 1 day at 37°C, the antibacterial activity of the cleavage product, as a measure of cleavage efficiency, was analyzed by determining the MIC, in accordance with the guidelines of the Clinical and Laboratory Standards Institute.

### Determination of intracellular concentration of AAC-released free antibiotic using LC-MS/MS.

P. aeruginosa PA14 bacteria were grown to an OD_600_ of approximately 0.4, washed with PBS, and fixed in 2% paraformaldehyde in PBS. RAW 264.7 macrophages were incubated with fixed P. aeruginosa PA14 bacteria, which were preincubated with 100 nM AAC in HB, as described above for the intracellular killing assay. Cell extracts were prepared by incubation in 75% acetonitrile (ACN) for 1 h, followed by lyophilization by evaporation under N2 (TurboVap; Biotage, Charlotte, NC), reconstitution in 100 μl of 50% ACN and 0.1% formic acid (FA), and filtration using a 0.45 μm glass fiber filter plate (Phenomenex, Torrance, CA).

The G2637 antibiotic was separated from cell extracts on an Acquity UPLC (Waters Corporation, Milford, MA) under gradient elution using a Phenomenex Kinetex XB C_18_ column (100 Å, 50 by 2.1 mm [internal diameter], 2.6-μm particle size). The column was maintained at room temperature. The mobile phase was water containing 0.1% FA (A) and acetonitrile containing 0.1% FA (B) at a flow rate of 0.4 ml/min. G2637 was eluted with a gradient of 2% to 90% B over 2 min, followed by 2 min decreasing to 2% B to re-equilibrate the column. The injection volume was 10 μl. The Triple Quad 6500 mass spectrometer (Ab Sciex, Framingham, MA) was operated in a positive-ion multiple-reaction-monitoring (MRM) mode. The G2637 antibiotic precursor (Q1) ion monitored was 452.9 *m/z* with declustering potential at 110 V, and the product (Q3) ion monitored was 267.2 *m/z* with collision energy at 40 eV. Two other product ions were also monitored as qualifiers, 398.4 *m/z* and 309.23 *m/z* with collision energy at 23 eV and 39 eV, respectively. The MS/MS settings were as follows: ion spray voltage, 5,500 V; curtain gas, 40 lb/in^2^; nebulizer gas (GS1), 35 lb/in^2^; GS2, 50 lb/in^2^; temperature, 600°C; and dwell time, 100 ms. Linear calibration curves were obtained for a 0.5 to 64 nM concentration range by spiking G2637 into cell and supernatant fractions (lacking P. aeruginosa and AAC) that were treated similarly to experimental samples. Concentrations of G2637 antibiotic were calculated with MultiQuant software (Ab Sciex) and expressed in picomoles per well (*C*_W_). For 26F8-cBuCit-G2637 AAC, concentrations in cell lysates were converted to estimated intracellular molar concentration of free G2637 antibiotic in macrophages (*C*_M_), based on a cell number of 3 × 10^5^ per well and an average cell radius of approximately 6 μm as determined by microscopy. The latter corresponds to an average spheric/cylindric volume of 0.55 × 10^−12^ liter/cell and a total macrophage volume of 1.65 × 10^−7^ (i.e., [3 × 10^5^] × [0.55 × 10^−12^]) liter per well, resulting in a conversion from *C*_W_ (picomoles per well) to *C*_M_ (micromolar units) as follows: *C*_M_ = 10^6^ × [*C*_W_/(1.65 × 10^−7^)].

### Statistical analysis.

The data were analyzed by unpaired *t* test to assess statistical differences.

## References

[1] Thaden JT, Park LP, Maskarinec SA, Ruffin F, Fowler VG, van Duin D. 2017. Results from a 13-year prospective cohort study show increased mortality associated with bloodstream infections caused by Pseudomonas aeruginosa compared to other bacteria. Antimicrob Agents Chemother 61:e02671-16. 28373189 10.1128/AAC.02671-16 PMC5444115

[2] Planquette B, Timsit J-F, Misset BY, Schwebel C, Azoulay E, Adrie C, Vesin A, Jamali S, Zahar J-R, Allaouchiche B, Souweine B, Darmon M, Dumenil A-S, Goldgran-Toledano D, Mourvillier BH, Bédos J-P. 2013. Pseudomonas aeruginosa ventilator-associated pneumonia. Predictive factors of treatment failure. Am J Respir Crit Care Med 188:69–76. 10.1164/rccm.201210-1897OC. 23641973

[3] Horcajada JP, Montero M, Oliver A, Sorlí L, Luque S, Gómez-Zorrilla S, Benito N, Grau S. 2019. Epidemiology and treatment of multidrug-resistant and extensively drug-resistant Pseudomonas aeruginosa infections. Clin Microbiol Rev 32:e00031-19. 10.1128/CMR.00031-19. 31462403 PMC6730496

[4] Cillóniz C, Gabarrús A, Ferrer M, de la Bellacasa JP, Rinaudo M, Mensa J, Niederman MS, Torres A. 2016. Community-acquired pneumonia due to multidrug- and non-multidrug-resistant Pseudomonas aeruginosa. Chest 150:415–425. 10.1016/j.chest.2016.03.042. 27060725

[5] Zilberberg MD, Shorr AF. 2013. Prevalence of multidrug-resistant pseudomonas aeruginosa and carbapenem-resistant enterobacteriaceae among specimens from hospitalized patients with pneumonia and bloodstream infections in the United States from 2000 to 2009: drug resistance in pneumonia and BSI. J Hosp Med 8:559–563. 10.1002/jhm.2080. 24022878

[6] Juan C, Peña C, Oliver A. 2017. Host and pathogen biomarkers for severe Pseudomonas aeruginosa infections. J Infect Dis 215:S44–S51. 10.1093/infdis/jiw299. 28375513

[7] Tacconelli E, Carrara E, Savoldi A, Harbarth S, Mendelson M, Monnet DL, Pulcini C, Kahlmeter G, Kluytmans J, Carmeli Y, Ouellette M, Outterson K, Patel J, Cavaleri M, Cox EM, Houchens CR, Grayson ML, Hansen P, Singh N, Theuretzbacher U, Magrini N, Aboderin AO, Al-Abri SS, Jalil NA, Benzonana N, Bhattacharya S, Brink AJ, Burkert FR, Cars O, Cornaglia G, Dyar OJ, Friedrich AW, Gales AC, Gandra S, Giske CG, Goff DA, Goossens H, Gottlieb T, Blanco MG, Hryniewicz W, Kattula D, Jinks T, Kanj SS, Kerr L, Kieny M-P, Kim YS, Kozlov RS, Labarca J, Laxminarayan R, Leder K, Who Pathogens Priority List Working Group, et al. 2018. Discovery, research, and development of new antibiotics: the WHO priority list of antibiotic-resistant bacteria and tuberculosis. Lancet Infect Dis 18:318–327. 10.1016/S1473-3099(17)30753-3. 29276051

[8] CDC. Antibiotic resistance threats in the United States. 2019. 10.15620/cdc:82532.

[9] Jorth P, Staudinger BJ, Wu X, Hisert KB, Hayden H, Garudathri J, Harding CL, Radey MC, Rezayat A, Bautista G, Berrington WR, Goddard AF, Zheng C, Angermeyer A, Brittnacher MJ, Kitzman J, Shendure J, Fligner CL, Mittler J, Aitken ML, Manoil C, Bruce JE, Yahr TL, Singh PK. 2015. Regional isolation drives bacterial diversification within cystic fibrosis lungs. Cell Host Microbe 18:307–319. 10.1016/j.chom.2015.07.006. 26299432 PMC4589543

[10] Chau CH, Steeg PS, Figg WD. 2019. Antibody-drug conjugates for cancer. Lancet Lond Engl 394:793–804. 10.1016/S0140-6736(19)31774-X. 31478503

[11] Lambert JM, Berkenblit A. 2018. Antibody-drug conjugates for cancer treatment. Annu Rev Med 69:191–207. 10.1146/annurev-med-061516-121357. 29414262

[12] Lehar SM, Pillow T, Xu M, Staben L, Kajihara KK, Vandlen R, DePalatis L, Raab H, Hazenbos WL, Morisaki JH, Kim J, Park S, Darwish M, Lee B-C, Hernandez H, Loyet KM, Lupardus P, Fong R, Yan D, Chalouni C, Luis E, Khalfin Y, Plise E, Cheong J, Lyssikatos JP, Strandh M, Koefoed K, Andersen PS, Flygare JA, Tan MW, Brown EJ, Mariathasan S. 2015. Novel antibody–antibiotic conjugate eliminates intracellular S. aureus. Nature 527:323–328. 10.1038/nature16057. 26536114

[13] Peck M, Rothenberg ME, Deng R, Lewin-Koh N, She G, Kamath AV, Carrasco-Triguero M, Saad O, Castro A, Teufel L, Dickerson DS, Leonardelli M, Tavel JA. 2019. A phase 1, randomized, single-ascending-dose study to investigate the safety, tolerability, and pharmacokinetics of DSTA4637S, an anti-Staphylococcus aureus thiomab antibody-antibiotic conjugate, in healthy volunteers. Antimicrob Agents Chemother 63:e02588-18. 10.1128/AAC.02588-18. 30910894 PMC6535527

[14] Cassin EK, Tseng BS. 2019. Pushing beyond the envelope: the potential roles of OprF in Pseudomonas aeruginosa biofilm formation and pathogenicity. J Bacteriol 201:e00050-19. 10.1128/JB.00050-19. 31010902 PMC6707909

[15] Fong R, Kajihara K, Chen M, Hotzel I, Mariathasan S, Hazenbos WLW, Lupardus PJ. 2018. Structural investigation of human S. aureus-targeting antibodies that bind wall teichoic acid. MAbs 10:979–991. 10.1080/19420862.2018.1501252. 30102105 PMC6204806

[16] Poxton IR. 1995. Antibodies to lipopolysaccharide. J Immunol Methods 186:1–15. 10.1016/0022-1759(95)00123-R. 7561138

[17] Pier GB, Thomas DM. 1982. Lipopolysaccharide and high-molecular-weight polysaccharide serotypes of Pseudomonas aeruginosa. J Infect Dis 145:217–223. 10.1093/infdis/145.2.217. 6798136

[18] Faure K, Shimabukuro D, Ajayi T, Allmond LR, Sawa T, Wiener-Kronish JP. 2003. O-antigen serotypes and type III secretory toxins in clinical isolates of Pseudomonas aeruginosa. J Clin Microbiol 41:2158–2160. 10.1128/JCM.41.5.2158-2160.2003. 12734267 PMC154700

[19] Rocchetta HL, Burrows LL, Pacan JC, Lam JS. 1998. Three rhamnosyltransferases responsible for assembly of the A-band D-rhamnan polysaccharide in Pseudomonas aeruginosa: a fourth transferase, WbpL, is required for the initiation of both A-band and B-band lipopolysaccharide synthesis. Mol Microbiol 28:1103–1119. 10.1046/j.1365-2958.1998.00871.x. 9680202

[20] Wei B, Gunzner-Toste J, Yao H, Wang T, Wang J, Xu Z, Chen J, Wai J, Nonomiya J, Tsai SP, Chuh J, Kozak KR, Liu Y, Yu S-F, Lau J, Li G, Phillips GD, Leipold D, Kamath A, Su D, Xu K, Eigenbrot C, Steinbacher S, Ohri R, Raab H, Staben LR, Zhao G, Flygare JA, Pillow TH, Verma V, Masterson LA, Howard PW, Safina B. 2018. Discovery of peptidomimetic antibody-drug conjugate linkers with enhanced protease specificity. J Med Chem 61:989–1000. 10.1021/acs.jmedchem.7b01430. 29227683

[21] Staben LR, Koenig SG, Lehar SM, Vandlen R, Zhang D, Chuh J, Yu S-F, Ng C, Guo J, Liu Y, FourieO’Donohue A, Go M, Linghu X, Segraves NL, Wang T, Chen J, Wei B, Phillips GDL, Xu K, Kozak KR, Mariathasan S, Flygare JA, Pillow TH. 2016. Targeted drug delivery through the traceless release of tertiary and heteroaryl amines from antibody–drug conjugates. Nat Chem 8:1112–1119. 10.1038/nchem.2635. 27874860

[22] Smith PA, Koehler MFT, Girgis HS, Yan D, Chen Y, Chen Y, Crawford JJ, Durk MR, Higuchi RI, Kang J, Murray J, Paraselli P, Park S, Phung W, Quinn JG, Roberts TC, Rougé L, Schwarz JB, Skippington E, Wai J, Xu M, Yu Z, Zhang H, Tan M-W, Heise CE. 2018. Optimized arylomycins are a new class of Gram-negative antibiotics. Nature 561:189–194. 10.1038/s41586-018-0483-6. 30209367

[23] Bentley AT, Klebba PE. 1988. Effect of lipopolysaccharide structure on reactivity of antiporin monoclonal antibodies with the bacterial cell surface. J Bacteriol 170:1063–1068. 10.1128/JB.170.3.1063-1068.1988. 2830227 PMC210874

[24] Storek KM, Auerbach MR, Shi H, Garcia NK, Sun D, Nickerson NN, Vij R, Lin Z, Chiang N, Schneider K, Wecksler AT, Skippington E, Nakamura G, Seshasayee D, Koerber JT, Payandeh J, Smith PA, Rutherford ST. 2018. Monoclonal antibody targeting the β-barrel assembly machine of Escherichia coli is bactericidal. Proc Natl Acad Sci U S A 115:201800043. 10.1073/pnas.1800043115. PMC5889671 29555747

[25] Storek KM, Chan J, Vij R, Chiang N, Lin Z, Bevers J, Koth CM, Vernes J-M, Meng YG, Yin J, Wallweber H, Dalmas O, Shriver S, Tam C, Schneider K, Seshasayee D, Nakamura G, Smith PA, Payandeh J, Koerber JT, Comps-Agrar L, Rutherford ST. 2019. Massive antibody discovery used to probe structure–function relationships of the essential outer membrane protein LptD. Elife 8:e46258. 10.7554/eLife.46258. 31237236 PMC6592684

[26] DiGiandomenico A, Warrener P, Hamilton M, Guillard S, Ravn P, Minter R, Camara MM, Venkatraman V, MacGill RS, Lin J, Wang Q, Keller AE, Bonnell JC, Tomich M, Jermutus L, McCarthy MP, Melnick DA, Suzich JA, Stover CK. 2012. Identification of broadly protective human antibodies to Pseudomonas aeruginosa exopolysaccharide Psl by phenotypic screening. J Exp Med 209:1273–1287. 10.1084/jem.20120033. 22734046 PMC3405507

[27] Sadikot RT, Blackwell TS, Christman JW, Prince AS. 2005. Pathogen–host interactions in Pseudomonas aeruginosa pneumonia. Am J Respir Crit Care Med 171:1209–1223. 10.1164/rccm.200408-1044SO. 15695491 PMC2718459

[28] Schweizer HP, Hoang TT. 1995. An improved system for gene replacement and xylE fusion analysis in Pseudomonas aeruginosa. Gene 158:15–22. 10.1016/0378-1119(95)00055-B. 7789804

[29] Hmelo LR, Borlee BR, Almblad H, Love ME, Randall TE, Tseng BS, Lin C, Irie Y, Storek KM, Yang JJ, Siehnel RJ, Howell PL, Singh PK, Tolker-Nielsen T, Parsek MR, Schweizer HP, Harrison JJ. 2015. Precision-engineering the Pseudomonas aeruginosa genome with two-step allelic exchange. Nat Protoc 10:1820–1841. 10.1038/nprot.2015.115. 26492139 PMC4862005

[30] Penterman J, Nguyen D, Anderson E, Staudinger BJ, Greenberg EP, Lam JS, Singh PK. 2014. Rapid evolution of culture-impaired bacteria during adaptation to biofilm growth. Cell Rep 6:293–300. 10.1016/j.celrep.2013.12.019. 24412364 PMC3941072

[31] Studier FW. 2005. Protein production by auto-induction in high-density shaking cultures. Protein Expr Purif 41:207–234. 10.1016/j.pep.2005.01.016. 15915565

[32] Kunkel TA. 1985. Rapid and efficient site-specific mutagenesis without phenotypic selection. Proc Natl Acad Sci U S A 82:488–492. 10.1073/pnas.82.2.488. 3881765 PMC397064

[33] Junutula JR, Raab H, Clark S, Bhakta S, Leipold DD, Weir S, Chen Y, Simpson M, Tsai SP, Dennis MS, Lu Y, Meng YG, Ng C, Yang J, Lee CC, Duenas E, Gorrell J, Katta V, Kim A, McDorman K, Flagella K, Venook R, Ross S, Spencer SD, Wong WL, Lowman HB, Vandlen R, Sliwkowski MX, Scheller RH, Polakis P, Mallet W. 2008. Site-specific conjugation of a cytotoxic drug to an antibody improves the therapeutic index. Nat Biotechnol 26:925–932. 10.1038/nbt.1480. 18641636

[34] Sawa T. 2014. The molecular mechanism of acute lung injury caused by Pseudomonas aeruginosa: from bacterial pathogenesis to host response. J Intensive Care 2:10. 10.1186/2052-0492-2-10. 25520826 PMC4267601

