# Supplementary figures and images for: Potent Killing of Pseudomonas aeruginosa by an Antibody-Antibiotic Conjugate
Source: mBio. 2021 Jun 1;12(3):e00202-21. doi: 10.1128/mBio.00202-21 (PMC8262897; doi:10.1128/mBio.00202-21)

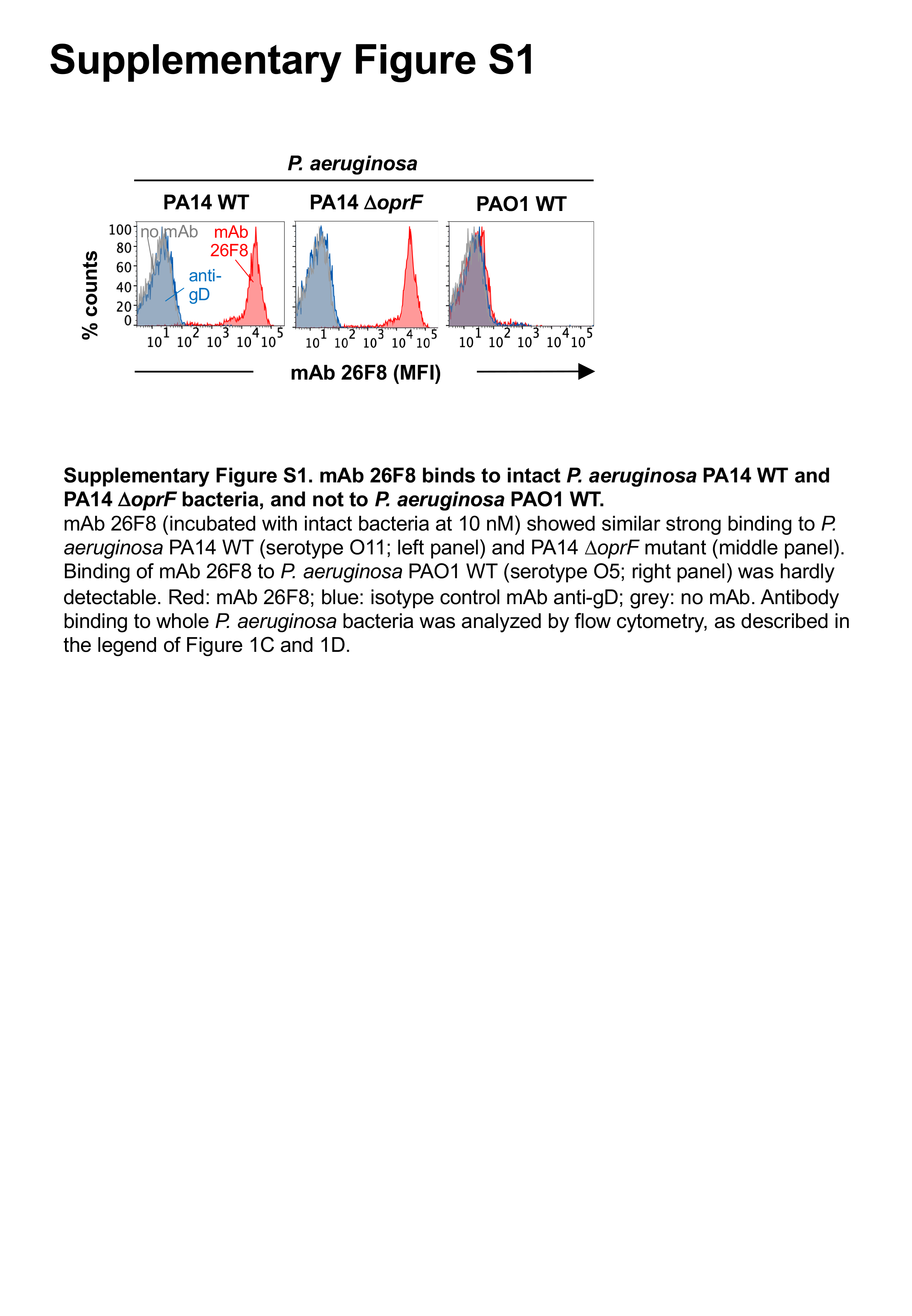

Supplement: FIG S1 [file mbio.00202-21-sf001.tif]

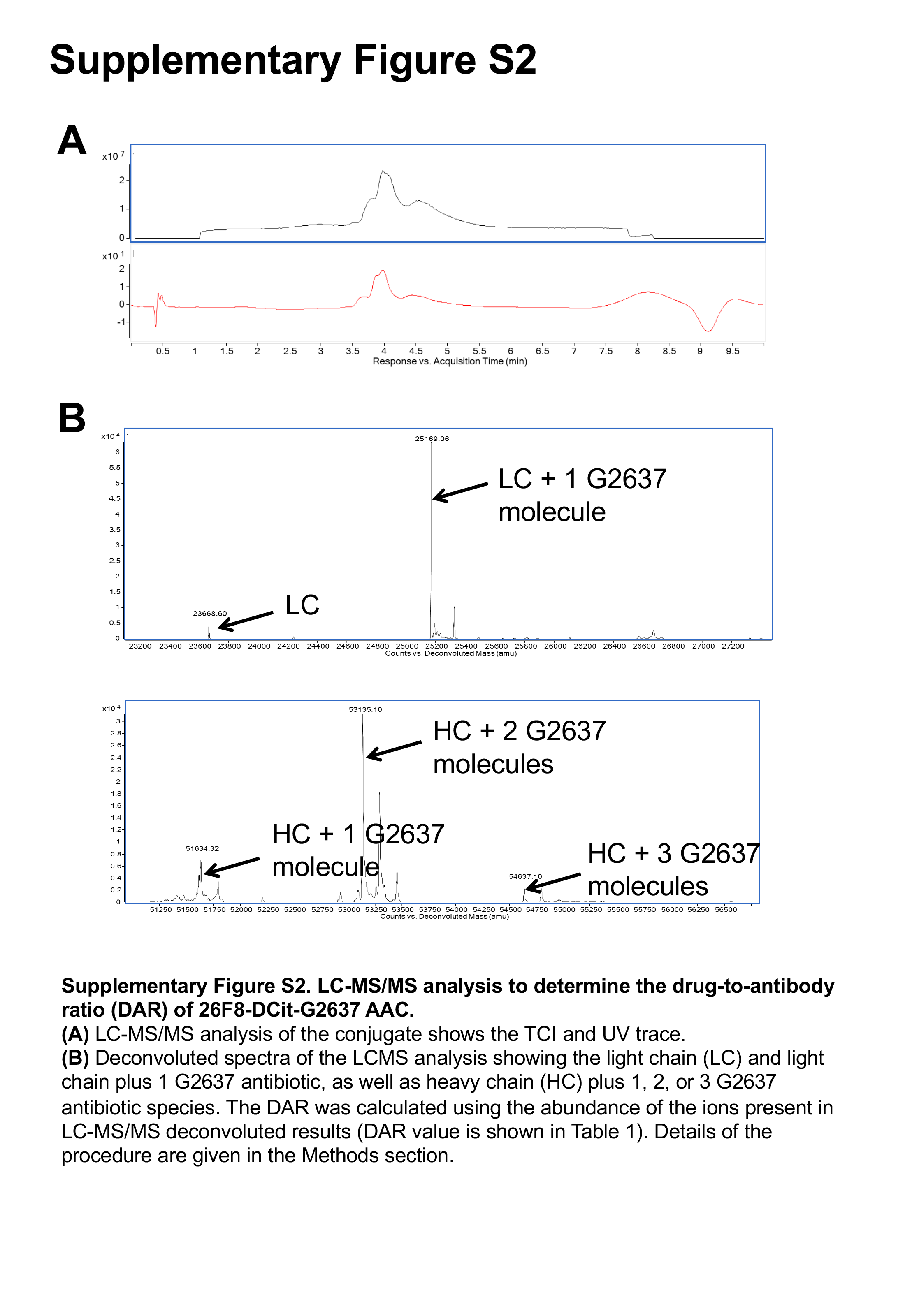

Supplement: FIG S2 [file mbio.00202-21-sf002.tif]

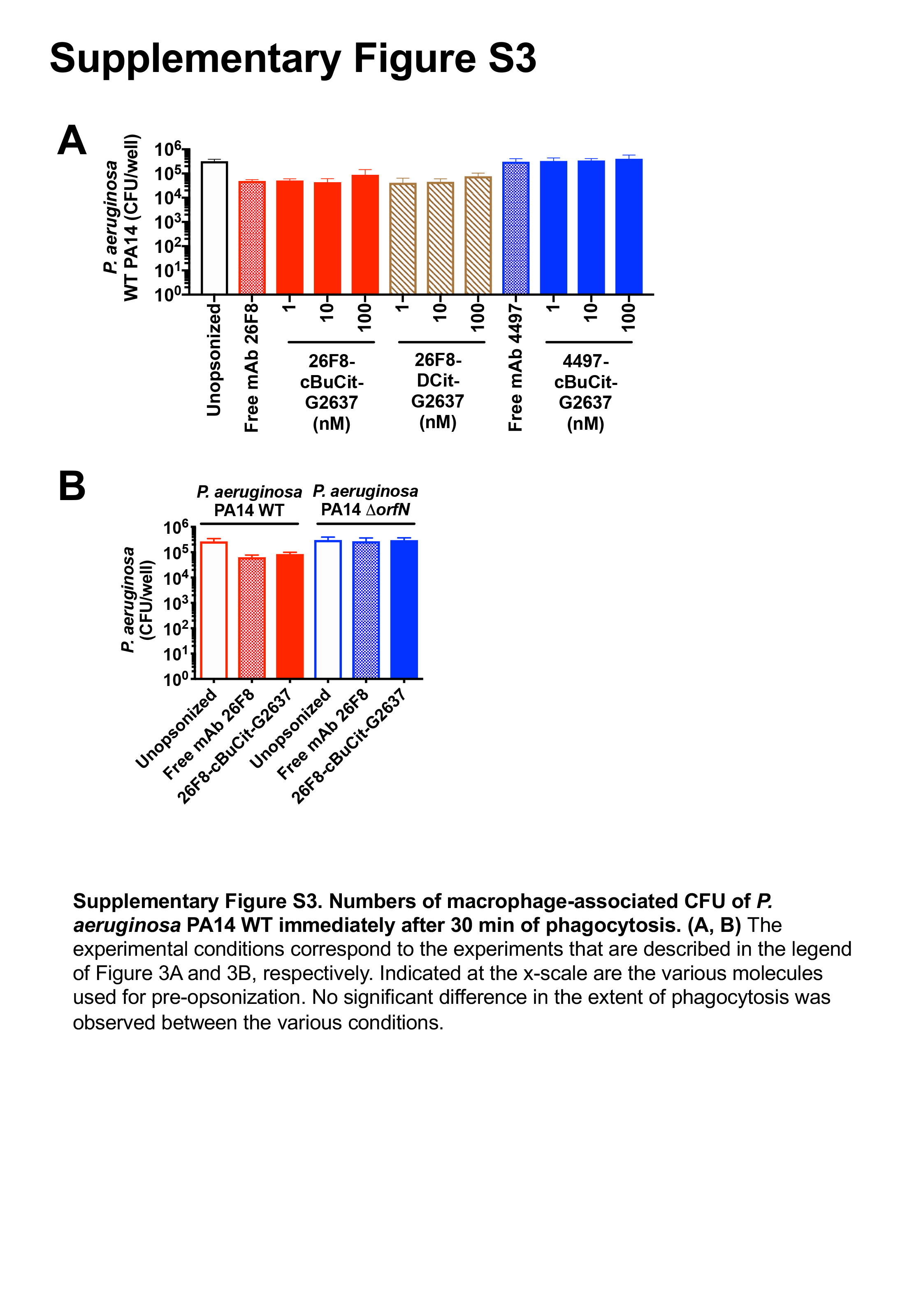

Supplement: FIG S3 [file mbio.00202-21-sf003.tif]

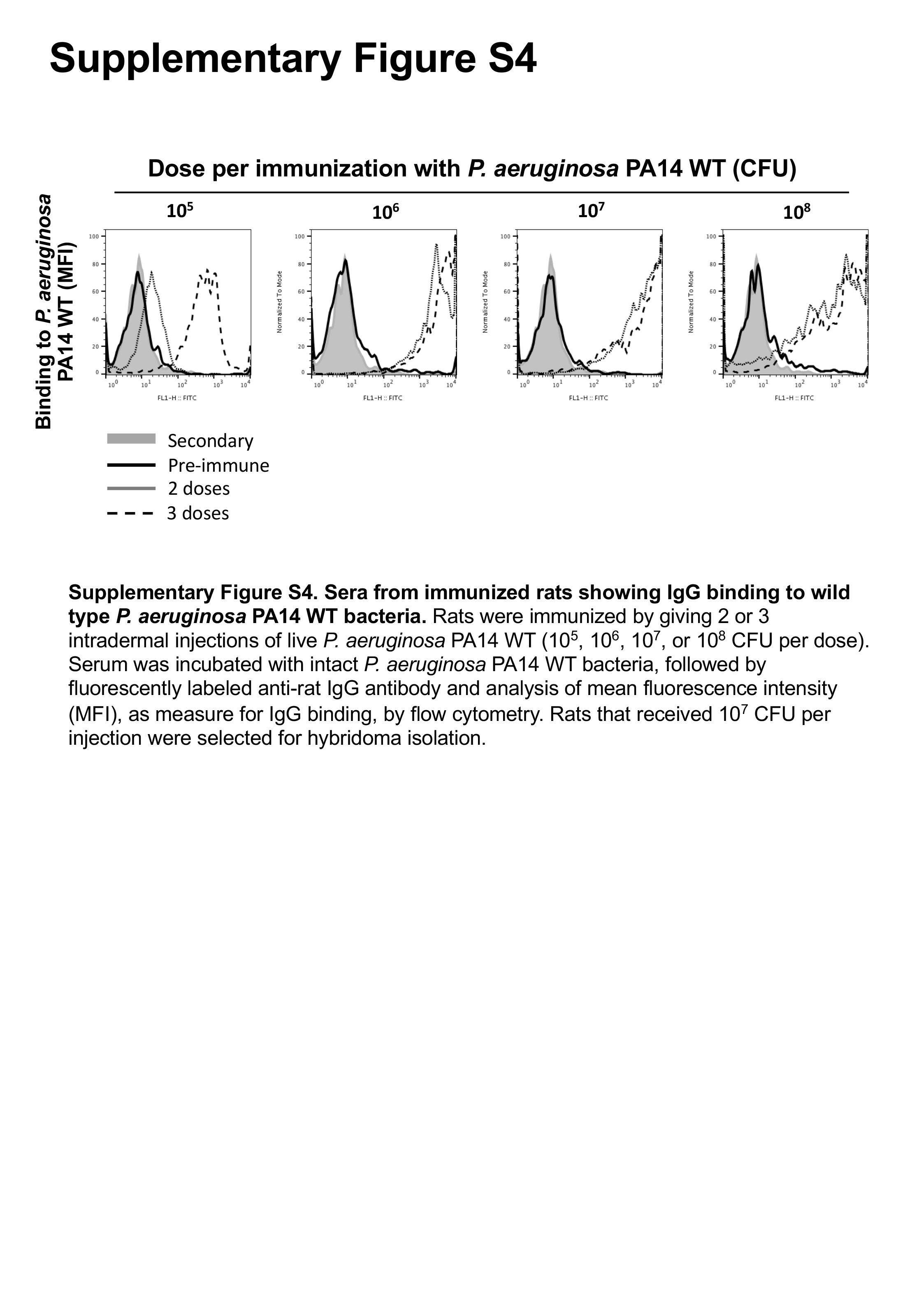

Supplement: FIG S4 [file mbio.00202-21-sf004.tif]
