# Supplementary material for: Potent Killing of Pseudomonas aeruginosa by an Antibody-Antibiotic Conjugate
Source: mBio. 2021 Jun 1;12(3):e00202-21. doi: 10.1128/mBio.00202-21 (PMC8262897; doi:10.1128/mBio.00202-21)
Supplement: TABLE S1 [file mbio.00202-21-st001.docx]

**Supplementary Table S1. MIC values of G2637 antibiotic for Gram-negative bacteria, as determined in MHB at pH 7 or pH 5.**

| Strain | MIC at pH 7 (μM) | MIC at pH 5 (μM) |
| --- | --- | --- |
| *Pseudomonas aeruginosa* PA14 WT | 2.0 + 0.0 | 1.7 + 0.6 |
| *Pseudomonas aeruginosa* PA14 *∆orfN* | 2.7 + 1.2 | 2.7 + 1.2 |
| *Escherichia coli* 25922 | 0.1 + 0.1 | nd |
| *Klebsiella pneumoniae* 43816 | 0.1 + 0.0 | nd |
| *Acinetobacter baumannii* 17078 | 1.3 + 0.5 | nd |

Minimum inhibitory concentration (MIC) values were determined using the standard protocol from the Clinical and Laboratory Standards Institute.

Shown are average values + SD of biological triplicates. Nd, not determined.
