# Supplementary material for: Potent Killing of Pseudomonas aeruginosa by an Antibody-Antibiotic Conjugate
Source: mBio. 2021 Jun 1;12(3):e00202-21. doi: 10.1128/mBio.00202-21 (PMC8262897; doi:10.1128/mBio.00202-21)
Supplement: TABLE S2 [file mbio.00202-21-st002.docx]

**Supplementary Table S2. Strains, plasmids, antibodies, primers, and DNA sequences used in this study.**

|  | **Genotype, description, or sequence** | **Source or reference** |
| --- | --- | --- |
| **Strains** | | |
| GNE230 | *P. aeruginosa* PA14 WT | (1) |
| GNE3963 | *P. aeruginosa* PA14 pBHR1-GFP | This study |
| GNE22 | *P. aeruginosa* PAO1 WT | ATCC 15692 |
| GNE310 | *P. aeruginosa* PA14 Δ*R2* Δ*orfN* (R2 pyocin and OrfN deletion) | This study |
| GNE3965 | *P. aeruginosa* PA14 Δ*oprF* | This study |
| GNE221 | *E. coli* S17-1 WT donor strain | ATCC 47055 |
| GNE231 | *E. coli* S17-1 pEX100T-*R2* pyocin deletion donor strain | This study |
| GNE298 | *E. coli* S17-1 pEX100T-*orfN* deletion donor strain | This study |
| GNE3905 | *E.coli* S17-1 pEX100T Δ*oprF* | This study |
| GNE5988 | *E. coli* pUCP19 *orfN* | This study |
| GNE6030 | *P. aeruginosa* Δ*R2*Δ*orfN* pUCP19 *orfN* | This study |
| GNE6033 | *P. aeruginosa* Δ*R2*Δ*orfN* pUCP19 empty | This study |
| BL21-DE3 | *E. coli* strain for protein expression | NEB (Ipswich, MA) |
|  | *E. coli* BL21-DE3 pET52b-ssHis8-OprF β-barrel | This study |
| **Plasmids** | | |
| pBHR1 | Broad range expression vector; the kanamycin resistance gene was replaced by gentamicin for efficient selection in *P. aeruginosa* | MoBiTec (Göttingen, Germany) |
| pET52b | Modified pET52b expression vector under control of the T7 lac promoter | This study |
| pET52b-ss-His8-OprF β-barrel | *E. coli* OmpA (residues M1-A21), followed by an N-terminal His8 tag, and the β-barrel domain of *P. aeruginosa* OprF (residues Q25-F184) subcloned into pET52b expression vector under the control of the T7 lac promoter | This study |
| pEX100T | Suicide vector for knockout of *P. aeruginosa* genes (*sacB*, *oriT*, pMB1) | ATCC 87436 (2) |
| pEX100T-Δ*oprF* | pEX100T used for deletion of *oprF* (locus PA14­_41570); *sacB* suicide vector used to delete *oprF* | This study |
| pEX100T-Δ*R2* | pEX100T-*R2* pyocin biosynthetic operon deletion (PA14_08020 to PA14_08150); *sacB* suicide vector used to delete R2 pyocin | This study |
| pEX100T-Δ*orfN* | pEX100T-*orfN* deletion (PA14_23460) *sacB* suicide vector used to delete *orfN* | This study |
| pUCP19 | *P. aeruginosa* expression vector | ATCC 87110 |
| pUCP19-*orfN* | *P. aeruginosa* expression vector expressing *orfN* | This study |
| **Primers used to generate and confirm gene knockouts (KO) in *P. aeruginosa* using Gibson Assembly technology (GA)** | | |
| pEX100T GA universal F | ggattaccctgttatccctag |  |
| pEX100T GA Universal R | gtagggataacagggtaatgag |  |
| R2 pyocin KO Up F GA | ctcattaccctgttatccctacGTATCCATTGCTTGCGGAATC |  |
| R2 pyocin KO Up R | GTAGGTGTGCTCCTGTTTAAGATGAGCCAGGC |  |
| R2 pyocin KO Dn F | CTCATCTTAAACAGGAGCACACCTACAGCTCCAG |  |
| R2 pyocin KO Dn R GA | ctagggataacagggtaatccCAATGTCGGGTTCCTGGAGC |  |
| R2 pyocin outside seq F | GTTGAGTTACGACTGGGCTG |  |
| R2 pyocin outside seq R | GGTGATCTGTAGCAAGCCAC |  |
| OrfN KO Up F GA | ctcattaccctgttatccctacCTCTCTAGCCGCTCGGGTTG |  |
| OrfN KO Up R | CTTTCCAGCTCGGGAAGCCAAAACCAGAAGTTC |  |
| OrfN KO Dn F | GTTTTGGCTTCCCGAGCTGGAAAGGCTATAGTTTGAGC |  |
| OrfN KO Dn R GA | ctagggataacagggtaatccCCGAGGAGCCTCATACGCAC |  |
| OrfN seq F | GTATGAAACATCCGGCTGCC |  |
| OrfN seq R | CAGAAAATGTAGCGCTACCGAG |  |
| **Primers used to amplify the *P. aeruginosa* PA14 *orfN* gene for cloning into pUCP19 plasmid** | | |
| *orfN* expression F | CCAAGCTTGCATGCCAAGGAGGTAAAAAATGAACTTCTGGTTTTGGCTTC |  |
| *orfN* expression R | CCTCTAGAGTCGACCCTATAGCCTTTCCAGCTCACCA |  |
| **DNA sequences synthesized for cloning** | | |
| *P. aeruginosa* *oprF* ORF (for synthesis of OprF for immunizations) | ATGAAAAAAACCGCCATCGCCATCGCCGTTGCACTCGCCGGATTCGCCACTGTCGCCCAAGCCCAAGGACAACATCACCATCACCACCATCACCATCAGGGTCAAAATAGTGTTGAAATTGAAGCCTTTGGTAAACGGTATTTCACGGATTCAGTTCGCAACATGAAAAACGCAGACTTATACGGAGGTTCTATTGGTTATTTCCTCACCGATGACGTTGAACTGGCATTGAGTTACGGAGAATATCATGACGTACGTGGTACATATGAAACGGGCAATAAAAAAGTGCATGGTAACCTGACCTCATTAGACGCCATTTATCATTTCGGGACACCTGGGGTTGGTTTGCGTCCATACGTTTCTGCGGGACTGGCACATCAGAACATTACCAACATCAACAGCGACTCCCAGGGACGCCAACAGATGACTATGGCGAATATCGGCGCAGGGCTCAAATATTATTTTACTGAAAATTTCTTTGCTAAAGCGTCCTTAGACGGTCAGTATGGGTTGGAAAAACGTGATAATGGACATCAAGGCGAATGGATGGCTGGATTGGGTGTGGGGTTTAATTTTTAA |  |
| 500 bp upstream of *P. aeruginosa* PA14 *oprF* (for *oprF* deletion construct) | AGGTGATGCTGAAAGTTTTGTACGGGTTGAAGAACTTTGAGGGCAAGTCGAAGTTCAAAACATGGCTATATAGCATCACGTACAACGAGTGCATCACGCAGTACCGCAAGGAGCGCCGCAAGCGCCGATTGATGGATGCGCTCAGCCTCGACCCGCTGGAGGAGGCTTCCGAGGAGAAGTCGCCGAAGGTTGAGGAGCGGGGCGGATTGGATCGTTGGCTCGTCCATGTCAATCCGATAGATCGGGAGATTCTGGTTCTTCGCTTCGTAGCGGAACTGGAGTTTCAGGAGATAGCGGATATCATGCACATGGGTCTGAGTGCGACGAAAATGCGCTACAAGCGGGCACTGGACCGCCTGCGCGAAAAGTTTTCAGATGCGACCGAAACATAGTTGGGTAAATATTGTCTCTCTATGCGGGAAGTTCTGATAAACTTGCCACCCAAGTTGTGCGGCTGATTGTTGGACAACTAACTGACCATCAAGATGGGGATTTAACGG |  |
| 500 bp downstream of *P. aeruginosa* PA14 *oprF* (for *oprF* deletion construct) | TCGGCTGAGCCTCTAAGGAAAAACCCGGCTCAGGCCGGGTTTTTCTTTGCCTGGAAAAAGACCGCTCGTCAGGCGCTCAGGGAAATCGGTTGCGACACGATGTCGCGGGTTACTTCGCCGATCACCAGGATCGCCGGGCTTTTCAAGGCGAAACGTCCGGCGTCCCGCAGTAGCTCGCCGAGGTTGCTGCGGCATTCGCGCTGGTTGCCCAAGGTGGCGTTCTCGATCATCGCCAGCGGCGTATCCTCGGCCATGCCTCCAGCCAGCAGCCCGGCCTGGATCTCCGCGAGTCGGGCGACGCCCATGTAGACCACCAGCGTGGTACCGCTGCGTGCCAGGGCTTCCCAGGCCAGCGGGCTGTCGTCCTGGGTATGCGCGGTCACCAGGGTCACGCCGCGGCTGATGCCGCGGTAGGTCAGCGGTATGCCGCAGGCAGTGGCGCCAGCCAGCCCGGCGGTGATGCCGTTCACTATCTCGCTGTCTATCCCGTGCCGGGCCAG |  |
| GFP | ATGACGGCATTGACGGAAGGTGCAAAACTGTTTGAGAAAGAGATCCCGTATATCACCGAACTGGAAGGCGACGTCGAAGGTATGAAATTTATCATTAAAGGCGAGGGTACCGGTGACGCGACCACGGGTACCATTAAAGCGAAATACATCTGCACTACGGGCGACCTGCCGGTCCCGTGGGCAACCCTGGTGAGCACCCTGAGCTACGGTGTTCAGTGTTTCGCCAAGTACCCGAGCCACATCAAGGATTTCTTTAAGAGCGCCATGCCGGAAGGTTATACCCAAGAGCGTACCATCAGCTTCGAAGGCGACGGCGTGTACAAGACGCGTGCTATGGTTACCTACGAACGCGGTTCTATCTACAATCGTGTCACGCTGACTGGTGAGAACTTTAAGAAAGACGGTCACATTCTGCGTAAGAACGTTGCATTCCAATGCCCGCCAAGCATTCTGTATATTCTGCCTGACACCGTTAACAATGGCATCCGCGTTGAGTTCAACCAGGCGTACGATATTGAAGGTGTGACCGAAAAACTGGTTACCAAATGCAGCCAAATGAATCGTCCGTTGGCGGGCTCCGCGGCAGTGCATATCCCGCGTTATCATCACATTACCTACCACACCAAACTGAGCAAAGACCGCGACGAGCGCCGTGATCACATGTGTCTGGTAGAGGTCGTGAAAGCGGTTGATCTGGACACGTATCAGTAA |  |
| **Antibodies** | | |
| 26F8 | Rat mAb against LPS O-antigen of *P. aeruginosa* PA14; with human IgG1 Fc tail | This study |
| 4497 | Human IgG1 mAb against βGlcNac modification of wall teichoic acid of *S. aureus* | (3, 4) |
| Anti-gD | Human IgG1 mAb against cytomegalovirus gD protein | (3) |
|  |  |  |

**REFERENCES**

1. Rahme L, Stevens E, Wolfort S, Shao J, Tompkins R, Ausubel F. 1995. Common virulence factors for bacterial pathogenicity in plants and animals. Science 268:1899–1902.

2. Schweizer HP, Hoang TT. 1995. An improved system for gene replacement and xylE fusion analysis in Pseudomonas aeruginosa. Gene 158:15–22.

3. Lehar SM, Pillow T, Xu M, Staben L, Kajihara KK, Vandlen R, DePalatis L, Raab H, Hazenbos WL, Morisaki JH, Kim J, Park S, Darwish M, Lee B-C, Hernandez H, Loyet KM, Lupardus P, Fong R, Yan D, Chalouni C, Luis E, Khalfin Y, Plise E, Cheong J, Lyssikatos JP, Strandh M, Koefoed K, Andersen PS, Flygare JA, Tan MW, Brown EJ, Mariathasan S. 2015. Novel antibody–antibiotic conjugate eliminates intracellular S. aureus. Nature 527:323–328.

4. Fong R, Kajihara K, Chen M, Hotzel I, Mariathasan S, Hazenbos WLW, Lupardus PJ. 2018. Structural investigation of human S. aureus-targeting antibodies that bind wall teichoic acid. Mabs 1–13.
