# Supplementary material for: Potent Killing of Pseudomonas aeruginosa by an Antibody-Antibiotic Conjugate
Source: mBio. 2021 Jun 1;12(3):e00202-21. doi: 10.1128/mBio.00202-21 (PMC8262897; doi:10.1128/mBio.00202-21)
Supplement: TEXT S1 [file mbio.00202-21-s0001.docx]

**SUPPLEMENTARY METHODS**

**Synthesis of linker-antibiotic molecule *cBuCit-G2637***

**Scheme:**

##### Experimental:

***General procedure for preparation of compound B:***

To a solution of *4-nitrophenyl carbonochloridate* (0.5 g, 2.48 mmol) and *2-(trimethylsilyl)ethanol* (0.35 g, 2.98 mmol) in DCM (10 mL) was added Et_3_N (0.5 g, 4.96 mmol) at 0 ^o^C, the mixture was stirred at 0 ^o^C for 1h. TLC (10% ethyl acetate in petroleum, R_f_ = 0.5) showed the reaction was completed. The mixture was poured into water (10 mL). The organic phase was separated and washed with brine (3 x 5 mL), dried over Na_2_SO_4_, filtered and concentrated to give the crude product, which was purified by flash chromatography on silica gel eluting with 1% EtOAc in petroleum ether to afford *4-nitrophenyl (2-(trimethylsilyl)ethyl) carbonate* (445 mg, 63.3%) as a white solid. ^1^H NMR (400 MHz, CD_3_OD): δ 8.27 (d, *J* = 8.8 Hz, 2H), 7.37 (d, *J* = 8.8 Hz, 2H), 4.40-4.36 (m, 2H), 1.17-1.13 (m, 2H), 0.08 (s, 9H).

***General procedure for preparation of compound 3:***

To a mixture of *4-nitrophenyl (2-(trimethylsilyl)ethyl) carbonate* (808.7 mg, 2.85 mmol) and *(S)-4-amino-2-(((benzyloxy)carbonyl)amino)butanoic acid* (600.0 mg, 2.38 mmol) in DMF (10 mL) was added Et_3_N (481.3 mg, 4.76 mmol) at 20 ^o^C. Then the reaction mixture was stirred for 12 h at 20 ^o^C, the TLC (10 % MeOH in DCM, Rf = 0.3) indicated the reaction was completed. The reaction mixture was acidified with aqueous HCl to pH = 5, which was extracted with EtOAc (2 x 30 mL). The organic phase was washed with brine (4 x 30 mL), dried over Na_2_SO_4_, filtered and concentrated to give the residue which was purified by flash chromatography on silica gel eluting with 10% methanol in ethyl acetate to afford Compound **3** (660 mg, 70%) as brown oil.

^1^H NMR (400 MHz, CD_3_Cl): δ 7.36-7.31 (m, 5H), 5.81-5.75 (m, 1H), 5.41 (brs, 1H), 5.11 (s, 2H), 4.43 (brs, 1H), 4.17-4.14 (m, 2H), 3.49 (s, 4H), 3.24 (br, 1H), 1.03-0.96 (m, 2H), 0.04 (s, 9H).

***General procedure for preparation of compound 2:***

To a solution of Compound **1** (100.0 mg, 0.12 mmol) in Ethanol (8 mL) was added 10% Palladium (12 mg) on carbon at 25 ^o^C. The reaction mixture was stirred for another 2 hours at 25 ^o^C under H_2_ atmosphere (15psi). The mixture was filtered and the filtrate was concentrated to dryness to obtained Compound **2** (84 mg, 99.8%) as a white solid. LCMS (5-95, AB, 1.5min): R_T_ (220/254 nm) = 0.680 min, m/z = 714.3[M+H]^+^.

***General procedure for preparation of compound 4:***

To a solution of Compound **3** (53.3 mg, 0.13 mmol) in DCM (5 mL) was added DIEA (26.1 mg, 0.20 mmol) and HATU (51.2 mg, 0.13 mmol), the mixture was stirred at 0 ^o^C for 20 min. Then to above mixture was added Compound **2** (48.0 mg, 0.07 mmol) in DMF (1mL) and stirred for another 16 hr at 20^o^C. The mixture was concentrated to remove DCM, and water (50 mL) was added. The mixture was filtered and the solid was dissolved in EtOAc (100 mL), washed with sat.aq Na_2_CO_3_ (30mL x 2), brine (30mL x 2), the organic was dried over Na_2_SO_4_, concentrated and purified prep-TLC (5% methanol in dichloromethane, R_f_ = 0.3) to give Compound **4** (50 mg, 68.1%) as a white solid. LCMS (5-95, AB, 1.5min): R_T_ (220/254 nm) = 1.066 min, m/z = 1115.8 [M+Na]^+^.

***General procedure for preparation of compound 5:***

To a solution of compound **4** (580.0 mg, 0.53 mmol) in Ethanol (20 mL) was added 10% Palladium (56.5mg) on carbon at 20 ^o^C. The reaction mixture was stirred for another 6 hours at 20 ^o^C under H_2_ atmosphere (15psi). The mixture was filtered and the filtrate was concentrated to dryness to obtained compound **5** (508 mg, 99.8%) as a white solid, which was used directly in the next step. LCMS (5-95, AB, 1.5min): R_T_ (220/254 nm) = 0.913 min, m/z = 958.5 [M+H]^+^.

***General procedure for preparation of compound 7:***

To a solution of compound **6** (180.9 mg, 0.64 mmol) in DCM (10 mL) was added DIEA (205.6 mg, 1.59 mmol) and HATU (241.9 mg, 0.64 mmol) at 20^o^C. The mixture was stirred at 20^o^C for 10min. Then to above mixture was added compound **5** (508.0 mg, 0.53 mmol) in DMF (1 mL) and stirred for another 1h at 20 ^o^C. The mixture was concentrated to remove dichloromethane and water (10 mL) was added. The mixture was filtered and the solid was dissolved in EtOAc (10 mL), washed with sat.aq Na_2_CO_3_ (10 mL x 2), brine (10 mL x 2), the organic was dried with Na_2_SO_4_, concentrated and purified by flash chromatography on silica gel eluting with 0-10% methanol in dichloromethane to give compound **7** (600 mg, 92.4%) as a white solid. LCMS (5-95, AB, 1.5min): R_T_ (220/254 nm) =1.212 min, m/z = 1224.9[M+H]^+^.

***General procedure for preparation of compound 8：***

To a solution of m compound **7** (600.0 mg, 0.49 mmol) in THF (6 mL) was added LiOH (41.0 mg, 0.98 mmol) in Water (2 mL). The mixture was stirred at 25 ^o^C for 2h. The reaction mixture was concentrated under reduced pressure to remove THF, then H_2_O (10 mL) was added, the mixture was acidified with 5% KHSO4 in water to pH = 5, taken up in EtOAc (20 mL). The organic was separated and washed with brine (2 x 10 mL). The organics was dried over Na_2_SO_4_ before concentration to dryness to give compound **8** (550 mg, 92.7%) as a white solid. LCMS (5-95, AB, 1.5 min): R_T_ (220/254 nm) =1.013 min, m/z = 1210.5 [M+H]^+^.

***General procedure for preparation of compound 9:***

A mixture of DIEA (117.5 mg, 0.91 mmol), *2-aminoacetonitrile hydrochloride* (54.7 mg, 0.59 mmol), compound **8** (550.0 mg, 0.45 mmol) in DCM (10 mL) was stirred at 0 ^o^C for 2min, then HATU (207.3 mg, 0.55 mmol) in DMF (1mL) was added to the above solution. The reaction mixture was stirred at 0^o^C for 2h. The mixture was concentrated to dryness and the residue was taken up in EtOAc (20 mL) and washed with H_2_O (20 mL), followed brine (2 x 10 mL). The organic layer was dried over Na_2_SO_4_ before concentration to dryness. The residue was purified by flash chromatography on silica gel eluting 8% methanol in DCM to give compound **9** (465 g, 79.5%) as a white solid. LCMS (5-95, AB, 1.5 min): R_T_ (220/225 nm) = 1.158min, m/z = 1249.8 [M+H]^+^.

***General procedure for preparation of compound 9:***

A solution of compound **9** (90.0 mg, 0.07 mmol) and TBAF (75 mg, 0.29 mmol) in DMF (5 mL) was stirred at 50^o^C for 1h. The reaction mixture was taken up in EtOAc (10 mL) and the organics washed with brine (4 x 10 mL). The organic layer was dried over Na_2_SO_4_, filtered and concentrated to give compound **10** (78 mg, 98%) as a white solid. The crude was used to next step directly. LCMS (5-95, AB, 1.5min): R_T_ (220 /254 nm) = 0.820 min, m/z = 1104.4 [M+H]^+^.

***General procedure for preparation of compound 12:***

To a solution of **compound 10** (34.0 mg, 0.03 mmol) in N,N-Dimethylformamide (2 mL) was added **compound 11** (34.0 mg, 0.05 mmol，*synthesis see J. Med. Chem. 2018, 989*) and DIEA (12.0 mg, 0.09 mmol). The mixture was stirred at 25^o^C for 1h. The mixture was concentrated and purified by Pre-TLC (10% MeOH in DCM) to give the product **compound 12** (85 mg, 92%) as a pale yellow solid. LCMS (5-95, AB, 1.5min): R_T_ (220/254nm) = 1.107 min, m/z = 801.4 [M-100/2+H]^+^.

***General procedure for preparation of cBuCit-G2637:***

A solution of **compound 12** (30.0 mg, 0.02 mmol) in 5% TFA / HFIP (3.0 mL) was stirred at 25 ^o^C for 2 hours. The mixture was concentrated in vacuo and purified by pre-HPLC (acetonitrile 37-57% / 0.075% TFA in water) to afford ***cBuCit-G2637*** (13.4 mg, 43.5%) as a white solid. LCMS (5-95, AB, 1.5 min): R_T_ (220/254 nm) = 0.710 min, m/z = 751.2 [M/2+H]^+^.
